# Promoting sleep and mental well-being in children: Protocol for a naturalistic pilot in-app study among users of the Aumio app

**DOI:** 10.1371/journal.pone.0322302

**Published:** 2025-04-29

**Authors:** Nadja Ruckser, Melissa Röcken, Jean Ochel, Alfred Wiater, Claudia Calvano

**Affiliations:** 1 Department of Education and Psychology, Division of Clinical Psychological Intervention, Freie Universität Berlin, Berlin, Germany; 2 Department of Psychiatry and Neurosciences, Campus Charité Mitte, Charité – Universitätsmedizin Berlin, Corporate Member of Freie Universität Berlin and Humboldt Universität zu Berlin, Berlin, Germany; 3 German Centre for Mental Health (DZPG), Berlin-Potsdam, Germany; 4 Aumio GmbH, Berlin, Germany; 5 Onlinepraxis für Kinderschlafmedizin, Bad Wiessee, Germany; 6 Department of Education and Psychology, Division of Clinical Child and Adolescent Psychology and Psychotherapy, Freie Universität Berlin, Berlin, Germany; Mae Fah Luang University School of Anti Aging and Regenerative Medicine, THAILAND

## Abstract

Sleep problems are widespread among children and can have a negative impact on their development, everyday functioning and pose an additional burden on the parents. While there are numerous apps designed to improve children’s sleep and help them fall asleep, little data are available regarding the feasibility, acceptability and efficacy. The purpose of this study is to conduct an initial analysis of the feasibility and acceptability of the Aumio app and an email psychoeducational module as well as preliminary efficacy on child’s sleep quality and the associated factors well-being and parental stress. This research project is designed as a single-group pre-post design for a sample of 456 guardian-child dyads, with children aged 6–12 years. Participants will be recruited through the Aumio app as well as through advertisements in pediatric clinics and online. During the 12-week intervention, participants will be provided with the Aumio app and the parent-centred email psychoeducation module. At the beginning of the intervention and before the first app use (T0), six weeks after the first app use (T1), and immediately following the conclusion of the 12-week intervention (T2), the feasibility, acceptability and preliminary efficacy on children’s sleep and health-related quality of life as well as parental stress will be examined. The study will examine the association between the intensity of the Aumio app use and the endpoints to improve feasibility and acceptability by synthesizing recommendations for use. This pilot study can provide important insights into an app targeting child’s sleep problems in naturalistic, uncontrolled settings. Through this study, the existing research gap regarding app-based interventions for improving infant sleep will be addressed. Results will stimulate further development and research in the area of evidence-based mobile health interventions for children and their parents.

## Introduction

Sleep problems are widespread among children in Germany, affecting more than 20% of children of preschool and primary school age [1]. The most common sleep problems in this age group are difficulties with falling or staying asleep, which may persist and remain for extended periods [2]. Simultaneously, sleep plays a crucial role in child development and is closely linked to physical, cognitive, behavioural, and social-emotional functions that affect children’s learning, behaviour, and overall well-being [3]. Furthermore, sleep is crucial for the development of complex executive, verbal and non-verbal cognitive functions [3]. Consequently, childhood sleep problems are associated with a variety of secondary impairments [4]. According to a recent review [3], a variety of detrimental outcomes are associated with insufficient sleep duration in children: insufficient sleep duration increases the risk of overweight or obesity and related health conditions in children. Moreover, insufficient sleep may be a risk factor for later development of ADHD and may lead to an increased occurrence of externalising behaviours such as hyperactivity, impulsivity, irritability, and conduct problems [5]. Other effects include daytime sleepiness and impaired daytime functioning, short attention span, and negative behavioural responses and interactions with peers [3]. Additionally, insufficient sleep, inadequate sleep (i.e., a shorter sleep duration than required to maintain daytime wakefulness), poor sleep quality, and especially sleepiness during the day are associated with problems in children’s learning, memory, and academic performance [6].

Given that sleep problems in toddlers and school-aged children can also negatively affect parents’ sleep, health, and daytime functioning [7], addressing parental burden in this context is particularly relevant. A meta-analysis by Varma, Conduit, Junge, Lee, and Jackson [8] found associations between child sleep problems and poorer sleep quality on side of the parents, more severe insomnia symptoms, and increased arousal before parents go to sleep. In addition, poor child sleep has been negatively associated with the parents’ partnership quality and between parent and child relations [9]. Therefore, addressing parental burden within interventions for child’s sleeping problems is a promising approach, not only for the parents themselves, but also with respect to possible influences on children’s therapy processes and outcomes [10].

A meta-analysis conducted by Zhu, Xiao, and Tu [11] covering 15 studies demonstrated that technology-based interventions can contribute to the improvement of children’s sleep by increasing the total sleep time, sleep efficiency as well as decreasing the sleep onset latency and time span awake after sleep onset. Although the effect sizes were generally small to moderate, the studies demonstrated good overall quality. However, there was high heterogeneity in the data and the types of technology used. Additionally, the majority of technology-based interventions targeted children’s caregivers by providing sleep behavior management strategies and parental stress was not addressed. While the six uncontrolled pre-post studies demonstrated significant mean effect size in all outcome indicators, the eight randomized controlled trials only showed effects in wake after sleep onset. However, the trials conducted so far included 5 different intervention types. Up to now, there is limited research on audio-based mobile applications for improving children’s sleep quality [12].

To date, the Aumio app has been evaluated in a few unpublished master’s theses. However, this marks the first scientific study to assess the Aumio app using a large sample size, following a preregistered study protocol, with the intention of disseminating the findings. We aim to investigate the feasibility and acceptability of the Aumio app and the email psychoeducational module. As secondary user-centred outcomes, preliminary efficacy on child’s sleep quality and associated factors will be investigated. This current trial aims to fill a gap in research by being the first to investigate the feasibility and application of a German-language audio based app for improving children’s sleep quality with a large sample in a naturalistic setting. Additionally, the study examines preliminary’ efficacy of the Aumio app on child’s sleep quality, health related quality of life and parental stress as well as the concurrent application of a parent-centred email psychoeducation module.

### Objectives

The aim of this pilot study is to test the feasibility, acceptability and preliminary efficacy of the Aumio app and the parent-centred email psychoeducation module. We aimed (1) to examine the feasibility and acceptability of the of the Aumio app and the parent-centred email psychoeducation module. We therefore aimed to conduct qualitative and quantitative data to gain comprehensive insights. In addition, we also aimed (2) to collect information for improvements of the Aumio app and the parent-centred email psychoeducation module from a user’s perspective. We furthermore aimed (3) to examine the preliminary efficacy of the Aumio app and the parent-centred email psychoeducation module. As secondary user-centred outcomes, we aimed to examine whether its use improves children’s sleep, covering a broad range of facets to adequately capture the multidimensional nature of sleep. Furthermore, preliminary efficacy on child’s health-related quality of life and effects on parental stress will be tested as secondary user-centred outcomes. In order to further improve the feasibility and to synthesize recommendations for use, it will be investigated (4) at which intensity of usage the Aumio app significantly affects the feasibility and satisfaction as well as the secondary user-centred outcomes. The intensity of usage will be analysed continuously as well as divided into 4 levels (12, 18, 24, and 30 uses during the study period) and included in the analyses.

The primary, feasibility hypothesis refers to the user’s subjective perspective on the Aumio app and the parent-centred email psychoeducation module, with the assessment time points 6 weeks after the start of the app use, at the midpoint of the 12-week parent-centred email psychoeducation module and again another 6 weeks after. We hypothesize that the Aumio app and the parent-centred email psychoeducation module is feasible. We also hypothesize Aumio app and the parent-centred email psychoeducation module is accepted among the users. We further hypothesize that the present study design is feasible to conduct a large scale trial on the effectiveness.

The secondary, user-centred hypothesis refer to comparisons of endpoint scores pre app use, at the same time points as the feasibility survey. We hypothesize that 6 weeks and 12 weeks after the start of the app use, children’s sleep quality will be significantly increased (secondary, child-centred endpoint) and health-related quality of life will be significantly higher (secondary, child-centred endpoint). Furthermore, we hypothesize that parenting stress will be significantly reduced 12 weeks after the start of the use of the email psychoeducational program (secondary, parent-centred endpoint). Exploratory analyses will also be conducted to determine to what extent usage behavior influences the effects on primary and secondary outcomes.

## Methods

### Ethics approval and consent to participate

Ethical approval and modification approval were granted by the Ethics Committee of the Department of Education and Psychology of Freie Universität Berlin on February 07, 2023, under reference number 047.2022.

### Trial design and study setting and participant timeline

The present research project is designed as a one-group pre-post pilot study. As this trial is the first larger scale pilot trial on the feasibility and acceptability as well as the first trial on the preliminary efficacy of the app on sleep quality and the parent module, randomization, control groups, and blinding do not apply to the study design of this trial. The study is a single study conducted in the naturalistic setting of the app, meaning it was conducted exclusively in-app and within a German-speaking population. After consenting to participate in this study, the participants will be provided a 12-months free access to the Aumio app and receive the parent-centred email psychoeducation module at weekly intervals.

**Fig 1:**
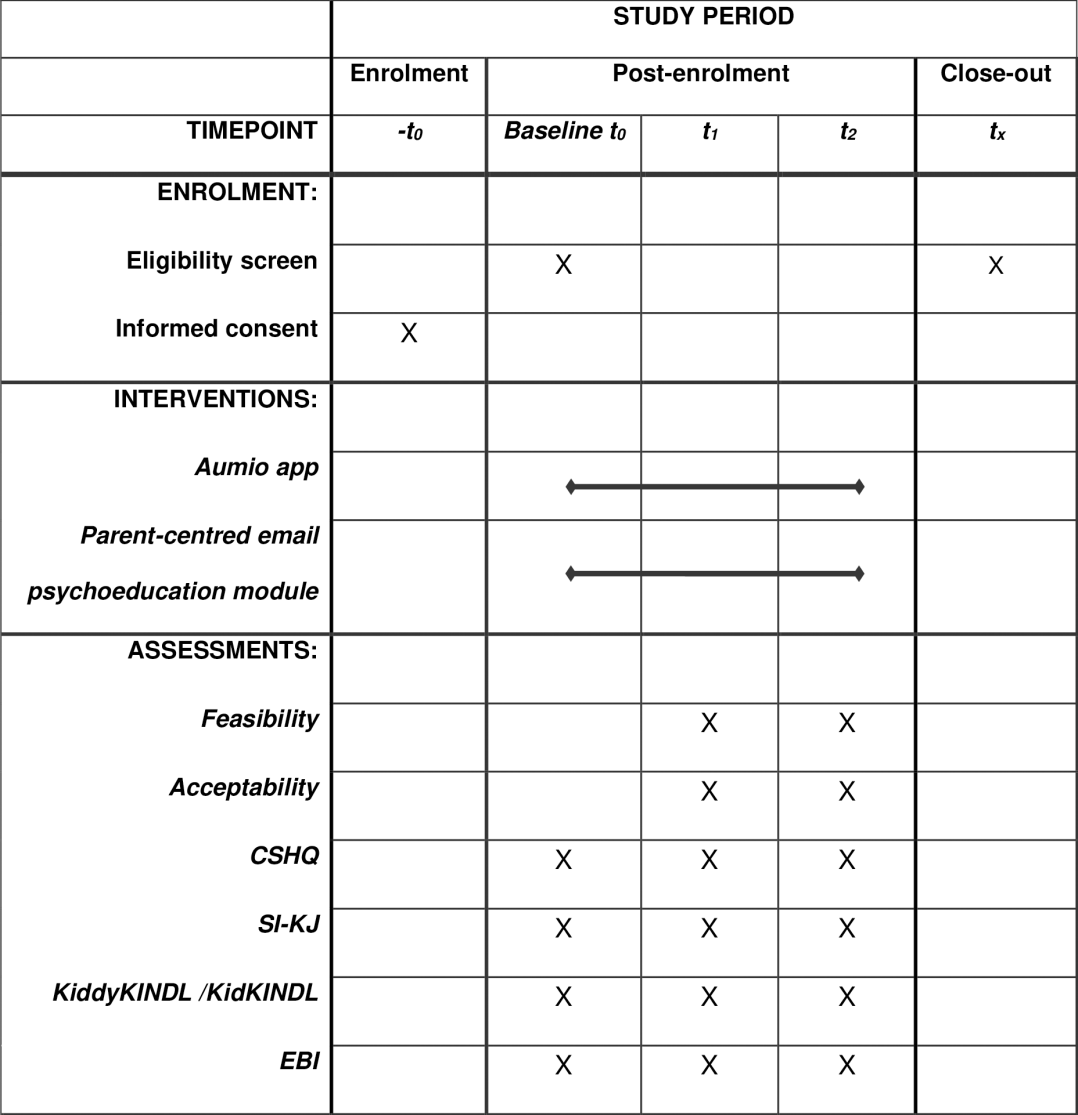
SPIRIT schedule of enrolment. CSHQ = Children’s Sleep Habits Questionnaire. SI-KJ = Sleep inventory for children (Schlafinventar für Kinder und Jugendliche), EBI = Parenting Stress Index (Eltern-Belastungs-Inventar).

Once participants agree to the conditions of participation and meet the inclusion criteria, guardians are directed to the first assessment (T0). At T0, at the beginning of the intervention and before the first app use, data on sociodemographic characteristics and relevant covariates and the outcomes will be collected. After completing the first survey T0, participants are provided with free access to the Aumio app for one year and receive the parent-centred email psychoeducation module once a week. The intervention duration is set at twelve weeks. Six weeks after the intervention start and the first app use, the second measurement point (T1) takes place, where primary and secondary outcomes are assessed. Immediately following the conclusion of the 12-week intervention, the third measurement point (T2) is conducted, comparable to the T1 assessment (Fig 1).

### Eligibility criteria

In line with the naturalistic setting of the study, the age range was set according to the target population of the Aumio app. Participants are eligible if the children are between 6 and 12 years old. The following exclusion criteria will be collected, assessed, and applied at T0: (1) insufficient German reading and understanding language proficiency of the child or guardian, (2) impaired hearing ability of the child, (3) diagnosed intelligence impairment or other severe developmental disability of the child, and (4) diagnosed obstructive sleep apnea of the child.

### Who will take informed consent?

The study participants (children and guardians) will receive written information about the purpose and procedure of the study and about possible advantages and disadvantages of participation. They will be informed that participation in the study is entirely voluntary, and that participation can be terminated at any time without giving reasons. Additionally, all participants will be informed about their rights regarding data storage, data protection and data deletion. Informed consent will be obtained from all guardians as well as children in child-friendly language prior to screening at T0. Only individuals who provide informed consent and child assent can participate in the study.

### Intervention description

The intervention consists of two components: the Aumio app and the parent-centred email psychoeducation module. Both are made available to participants. Aumio is a smartphone application designed to provide easily accessible health interventions for children and to promote healthy sleep and strengthen children’s mental health. It focuses on children’s sleep, relaxation, concentration and emotions. In the sleep module, Aumio offers a variety of audio stories, fantasy journeys and sounds for falling asleep to make going to bed in the evening as relaxing as possible for children. In addition, psychoeducation and the playful learning of relaxation techniques by means of meditations and dream journeys are integrated into the Aumio app. The meditation techniques are taught in a child-friendly way and made easily accessible for children. The exercises and stories are based on cognitive-behavioral techniques such as autogenic training or progressive muscle relaxation. The meditations and dream journeys for falling asleep have a duration between 5 and 20 minutes. In the intervention, families are advised to use the Aumio app at least twice a week in the evening, before bedtime. However, participants are encouraged to integrate the Aumio app into their daily routine in a manner that is suitable for them.

In addition, the parent-centred email psychoeducation module accompanies the use of the Aumio app and is emailed to the guardians once a week. The course addresses various aspects of children’s sleep to provide guardians with an understanding of child sleep and associated challenges. Each week focuses on a specific topic that covers relevant aspects of children’s sleep and sleep habits. First, the importance of good sleep quality for child development is discussed. Then, falling asleep is addressed by teaching the creation and establishment of bedtime routines and highlighting the importance of a comfortable sleep environment. Potential obstacles are also addressed. The parents are also taught how to help their children fall asleep on their own and how to make the process of accompanying their child to sleep less stressful. The course then tackles issues with staying asleep and provides strategies to support the child in falling back asleep after a nighttime awakening. Last, the course discusses how the child’s daytime activities can influence their sleep. For each topic, practical tips for implementing the concepts in daily life are provided alongside the theoretical explanations. The knowledge learned can be tested weekly in a small quiz in the Aumio app. In addition, the most important content is compiled in a child-friendly way and sent as a playfully designed info sheet as an attachment to the e-mail.

### Criteria for discontinuing or modifying allocated interventions

Participants can discontinue their use of the Aumio app, stop receiving the parent-centred email psychoeducation module, and consequently withdraw from the study at any time by uninstalling the app from their mobile devices and opting out of the email list. The intervention does not require contact with the study team.

### Strategies to improve adherence to interventions

The use of Aumio is entirely app based, and the meditations and dream journeys last between 5 and 20 minutes. Thus, the effort required to engage with the app can be considered minimal. As the naturalistic context of the study is among users of the Aumio app, the app functions are not altered for the study. Consequently, no reminders of app use will be used.

### Outcomes

#### Primary, feasibility outcome.

Feasibility will be assessed by the number of users, dropout rates, and usage patterns (e.g., number of audio content played). Usage behavior of the parent-centred email psychoeducation module will be assessed weekly using a content related quiz question in the Aumio app. The recruitment process will further be examined by analysing the study outreach method and its consequences on participant recruitment.

The acceptability of the Aumio app and the parent-centred email psychoeducation module will be assessed both quantitatively and qualitatively by examining the user’s satisfaction. The questions are derived from a study by Hiscock et al. [13], which examined the effects of a digital sleep intervention on child sleep and psychosocial outcomes of children and their caregivers. Satisfaction is assessed on 5-point Likert scale (1 = totally disagree/very unsatisfied, 5 = totally agree/very satisfied) generally and by specific factors, including satisfaction with the use of the product (“The app was easy to use”; “The e-mail course was understandable.”), the information provided (“The amount of information in the app was just right.”; “I was satisfied with the content provided by the app.”; “The amount of information in the e-mail course was just right.”; “I was pleased with the strategies the email course provided for my child’s sleep problem”; “I would have preferred to have a personal contact instead of the e-mail course to explain the strategies and their application.”), the feeling of safety (“I felt safe using the app.”; I felt safe participating in the email course.”), the subjective perception of improvement in the child’s sleep (“Using the app has helped my child to fall asleep.”; “Taking part in the e-mail course has helped my child to fall asleep better.”), and the intention to continue using the product in the future (“I would like to continue using the e-mail course in the future.; “I would like to use the app in the future.”). Likelyhood of recommendation to other parents (“How likely is it that you will recommend the Aumio app to other parents you know?”, “How likely is it that you will recommend the e-mail course to other parents you know?”) will be assessed using a 10-point Likert scale (1 = unlikely, 10 = highly likely). Quantitative data will be further supplemented by qualitative data on helpful content (“What has helped you and your child the most with the Aumio app?”, “What did you and your child find particularly helpful about the email course?”), ideas for improvement (“What would you change to improve the Aumio app?”, “What would you change to improve the email course?”), as well as moments when users experienced difficulty using the app (“When did you find it difficult to use the Aumio app?”). Additionally, we will assess changes in sleep duration (“Has your child’s sleep duration changed with the use of Aumio and the e-mail course?”) and whether parents sought advice from their local paediatrician regarding sleep problems (“While using the Aumio app and email course, did you contact your local paediatrician for more information on how to help your child sleep?”).

#### Secondary, user-centred outcomes.

For the assessment of child’s sleep quality, we used the Children’s Sleep Habits Questionnaire (CSHQ, 14]). The CSHQ is a retrospective screening instrument designed to examine children’s sleep [14]. It consists of 48 items, with 33 items distributed among various subscales, including bedtime problems, difficulties in falling asleep, sleep duration, anxiety, nighttime awakenings, parasomnias, breathing disorders, and daytime sleepiness. These subscales are utilized to calculate an overall score, providing a comprehensive evaluation of children’s sleep. Additionally, bedtime, the child’s average amount of sleep per day, duration of nocturnal wakefulness, and morning awakening time are recorded [14]. The German adaptation of Schlarb [15] is used, which was validated and normed by Schlarb, Schwerdtle, and Hautzinger [16] among a German norm sample consisting of 298 parents of 4–10-year-old children and 45 parents of children with sleep disorders.

To cover more aspects of sleep quality, we amended the CSHQ [14–16] by the Sleep inventory for children (Schlafinventar für Kinder und Jugendliche, SI-KJ; [17]). The retrospective, 33-item screening instrument supplements the survey by assessing sleep hygiene and sleep environment characteristics as well as physical factors contributing to sleep problems [17]. The questionnaire contains the main scale sleep and daily behaviour and the subscales sleep onset and sleep through problems, vegetative symptoms, daytime sensitivity, and nocturnal events. Data on various characteristics of sleep hygiene and sleep environment as well as physical factors, e.g., health problems such as allergies or chronic diseases are assessed. The instrument has been validated and normed on a German norm sample of 5–11-year-old children and parents [17].

We used the German version of the KiddyKINDL and KidKINDL, retrospective 24-item instruments to assess the health-related quality of life [18,19]. The KiddyKINDL is administered to guardians of 6-year-old children, and the KidKINDL is administered to guardians of 7–12-year-old children. Contents of the questionnaires are the subscales physical well-being, psychological well-being, self-esteem, family, friends, and functioning in daily life (school or preschool/kindergarten), with 4 items each [18,19]. Both versions are publicly available, have been psychometrically tested [20] and German normative scores are available [21].

To assess parenting stress, we used the German version of Abidin’s Parenting Stress Index (Eltern-Belastungs-Inventar, EBI; [22,23]). According to the retrospective 48-item instrument, parenting stress occurs when there is a mismatch between the demands of the parenting role and the coping resources needed to meet them. The demands arise from different sources of stress (child-related, parent-related and context-related), which are assigned to the child domain and the parent domain within the questionnaire. The child domain includes 5 subscales to assess the characteristics and behaviors of the child that result in demands on the parents, with 4 items for each subscale. This includes specific behavioral dispositions of the child (distractibility/hyperactivity, adaptability, demandingness, and mood), as well as interactions between child and parental characteristics (parental acceptance). The parenting domain includes 7 subscales to assess limitations in parental functioning that affect the resources available to cope with the demands of parenting and caring for children, with 4 items per subscale. This includes specific behavioral dispositions of the parents (depression, parental competence and parental attachment), as well as their available resources (social isolation, partner relationship, health and personal limitations). The instrument has been validated on German samples of 0.5-21-year-old children and their parents and German normative scores for mothers aged between 20 and 53 years are available [23]. For this study, parenting stress will be explored using the total score. In an exploratory manner, the 5 subscales of the child domain and the 7 parental domains will be analysed separately as well.

### Sociodemographic variables and covariates

At T0, sociodemographic characteristics and relevant covariates that have been identified in advance through a literature review are collected, including household size (“How many people live in your household all the time, including yourself?”, “Do other children live in your household besides the child with whom you are participating in our study?”; [24]), parental education (“What is your highest educational achievement?”; [24]), parental income (“Approximately what is the total average monthly net income of your household?”; [25]), relationship conflict between parents and parental separation (“Which marital status describes you best?”, “How would you describe your satisfaction with your relationship?”; [26,27]), sleeping together in the same bed (assessed through the SI-KJ; [28], child media use before bedtime (“How often does your child use electronic media before sleeping?”, “What electronic media does your child use before sleeping?”; [29]), and presence of media in the child’s sleep environment (“Is there electronic media in your child’s room?”, “What electronic media is in your child’s room?”; [30]). Covariates are assessed by categorial variables.

### Sample size

With regard to the required sample size, two factors were considered in the present study: The Aumio app for the children and the parent training. For the parent training, we first considered effects on child outcomes as a basis for sample size calculation. An average effect size of parent training of d = 0.20 was found in a meta-analysis on the effectiveness of family-based prevention interventions [31]. An average effect size of d = 0.21 of universal prevention programs was found in a meta-analysis on German-language effectiveness research [32]. Based on these findings, with an assumed power of.90, an alpha error level of.05, and an effect size of.21, a required sample size of 196 was calculated using the software g*Power [33].

Considering the drop-out rate (DOR) observed in the unpublished master’s theses on the Aumio app, this was also considered for sample calculation. In one of the most recent master’s theses on the effectiveness of the Aumio app, a drop out of 57.14% was found in the treatment group. Based on an expected DOR of 0.57, the number of cases is multiplied by the correction factor 1/(1-DOR) [34]. If the previously calculated sample size is increased by the expected DOR, a final required sample size of 456 is needed.

### Recruitment and treatment allocation

Participants are recruited and allocated consecutively. Study participants are mainly recruited through the Aumio app. After downloading the app in the app store, five percent of new users are offered the chance to enrol in the study before they start using the app for the first time. Recruitment will be augmented by advertisements in pediatric clinics and online, where a link to the website in the survey platform for informed consent is provided. If parents are interested in the study, they will be redirected to a dedicated page within the app. On this page, both children and guardians receive information about the opportunity to participate in the study. Participants are informed about the purpose and process of the survey, as well as possible advantages and disadvantages of participating. They will be informed that participation in the study is completely voluntary and that participation can be terminated at any time without giving reasons. Furthermore, participants will be informed about their rights regarding data storage, data protection and data deletion. Informed consent will be obtained from both guardians and children in child-friendly language.

### Plans to promote participant retention and complete follow-up

In return for participating, participants will receive free access to the entire Aumio app for one year. They also receive a 30 euro shopping voucher for completing the last survey (T2).

### Relevant concomitant care permitted or prohibited during the trial and Provisions for post-trial care

Participation in the current study does not affect any potential concomitant treatment during the study period. All participants will receive contact information of the study team and will be able to access evidence-based informational materials on infant sleep through the Aumio website at any time.

### Data collection and management

#### Plans for assessment and collection of outcomes.

The collection of the questionnaire data will be done using the German server of Tivian GmbH (UniPark), which specialises in the secure collection of data in the field of research and is certified with ISO 27001 by the German Federal Office for Information Security.

#### Data management.

The data of the questionnaires and the data of the app are collected separately, and the assignment is only possible via an assignment list, which are exclusively encrypted and password-protected and stored locally in separate folders. The hard drive will be located at Freie Universität Berlin.

No sensitive, personal data is collected via the Aumio app, but only data that has a technical background (e.g., device identification and information, language, etc.) and represents user behaviour (e.g., number and duration of app use). Furthermore, the data is only collected under a pseudonym, which is created by the participants themselves at T0. The pseudonym consists of the first two letters of the participant’s first name, the last two digits of their current ZIP code, and the first two letters of the name of the participant’s first child.

#### Confidentiality.

The data is only accessible to the study team. In addition, the data from Unipark and the data from the survey are assigned via an assignment list. This is password-protected, encrypted and only accessible to the study team. The data obtained are merged and then analysed completely anonymously. It is not possible to draw conclusions about individual persons. The data are only evaluated as a whole. All data are only stored locally or on a hard disk (as a back-up) in a password-protected manner. The required password is only accessible to the study team. The study team agrees to maintain confidentiality about personal data and to comply with the Federal Data Protection Act. No personal data will be passed on to third parties or published.

### Statistical methods

#### Statistical methods for primary and secondary outcomes.

Descriptive methods including the calculation of appropriate summary measures of the empirical distribution (mean, standard deviation, median, minimum and maximum for continuous variables, and frequency in percentages for categorical variables) and specification of missing observations are used to present socio demographic data, feasibility, acceptability (with the Aumio app and the email course) and data on app use. In addition to the quantitative analysis of feasibility and acceptability, open-ended responses will be examined for recurring themes and categorized in a structured manner to provide additional context and insight into user experiences.

Preliminary intervention effects on the outcomes are analyzed by repeated measures ANOVA. Furthermore, the study will explore the relationship between the intensity of use and the outcomes. This analysis will involve both app usage as continuous measure, and categorization of the intensity of use into four levels (e.g., 12, 18, 24, and 30 uses during the intervention period). We will assess whether significant effects on the outcomes are observed at different levels of app use. All statistical analyses will be performed using R.

#### Interim analyses.

No interim analyses on treatment effectiveness will be conducted

### Methods for additional analyses

Additional analyses will be conducted to examine whether effects differ between groups with different sociodemographic characteristics, e.g., household size, parental education, parental income, relationship conflict between parents, parental separation, co-sleeping and media use. We will therefore evaluate the effects of including sociodemographic characteristics as covariates in an analysis of covariance (ANCOVA).

### Methods in analysis to handle protocol non-adherence and any statistical methods to handle missing data

Multiple imputation methods are used to estimate missing data. The trial is supervised by the evaluation team, which is the contact for reporting of any adverse event.

#### Adverse event reporting and harms.

We expect no adverse events, as the interventions and assessment tools imply neither burden nor risks. However, in case of spontaneously reported adverse events like increased psychological burden, the families can contact the study team at any time and in case any adverse event is reported, it will be followed up and necessary steps will be taken.

#### Frequency and plans for auditing trial conduct.

No audits are planned

#### Plans for communicating important protocol amendments to relevant parties.

Any deviations from or changes to the protocol will be documented. Significant changes will be reported to the Ethics Committee of the Freie Universität Berlin and documented in the German Clinical Trials Registry.

#### Dissemination plans.

The results of the study will be submitted for publication in relevant journals and on the Aumio website.

## Discussion

The aim of the present pilot study is to test the feasibility, acceptability and preliminary efficacy of a German-language audio-based app for improving child sleep quality in combination with a parent-centred email psychoeducation module. Results will inform a future randomized-controlled clinical trial and for future modifications of the interventions. The significance of the present study lies in the pressing need for research in the field of mhealth interventions targeting child sleeping problems. While there are a high number of apps available that claim to help children in falling asleep and improving their sleep, most only offer white noise or soothing music and do not specifically address sleep habits [35]. Moreover, there is a scarcity of evidence-based and app-based interventions for children in general. Many apps promote improved sleep onset, well-being, and sleep habit development without incorporating evidence-based behavioural strategies [35]. Filling this gap using a large sample is a strength of the present study.

A limitation of the study design is the way in which the secondary user-centred objectives are collected. These are exclusively collected retrospectively in the third-party report by one parent. In particular, children’s sleep quality is not recorded in self-report. This approach may overlook potential cases of sleep problems in children, as highlighted by Paavonen et al. [36], who suggest that one-third of such cases may go undetected if only parents are surveyed. Therefore, it would have been beneficial to include both children’s and guardians’ perspectives in assessing children’s sleep. Due to the target group of children aged 6–12 years, sufficient reading skills are not to be expected in a comparatively large number of cases until the age of 7 or 8 years. Another limitation is the use of subjective, parent reported methods for assessing children’s sleep, without incorporating objective measures. Subjective methods for data collection depend on self-reported information and may be influenced by biases or inaccuracies [37]. Objective methods that directly measure sleep and recovery function and thus provide indirect information about sleep quality were not used [37]. In particular, discrepancies in the recording of total sleep time and wake after sleep onset have been reported in several studies [38]. This inconsistency must be considered when interpreting the data and highlight the need for a comprehensive approach that combines subjective and objective measures to obtain a more accurate understanding of sleep. Future studies should consider incorporating children’s self-report and guardians report as well as objective measures of sleep to provide a more comprehensive assessment of sleep quality.

Lastly regarding the analysis of the preliminary efficacy, the study design of the present research project, the single-group pre-post design, must be considered critically in itself. The methodological weaknesses were summarised by Maier-Riehle and Zwingmann [39]. First, the effect sizes cannot be interpreted causally because the observed changes are not exclusively attributed to the intervention but also to other confounding causes. In addition, different calculation methods are proposed for determining effect sizes in the single-group pre-post design. As a result, effect sizes are not comparable among each other and lead to different patterns of results. Last, it should be noted that Cohen’s limits of interpretation cannot be invoked when interpreting effect sizes, as these are based on independent samples and provide an overly optimistic measure in designs without a control group. No alternative limit of interpretation for effect sizes in single-group pre-post designs has been established at this time [39]. Nevertheless, the inclusion of covariates which possibly influence the primary and secondary outcomes aims to provide a more detailed picture of the preliminary efficacy of the Aumio app and can further prepare a larger, RCT with the focus on testing the efficacy of the Aumio app.

## Conclusion

In conclusion, the present pilot and feasibility study has the potential to address the existing research gap regarding app-based interventions for improving infant sleep. Through this study, insights into the feasibility and acceptability can be gained. Furthermore, initial insights on preliminary efficacy of such interventions in real life settings can be gained and a foundation for future work with more advanced and controlled study designs can be laid.

## Supporting information

S1 File SPIRIT Checklist. (DOCX)

S2 File Study_protocol. (DOCX)

S3 File Study_protocol_german. (DOCX)

## Abbreviations

DOR: Drop-Out Rate
CSHQ: Children’s Sleep Habits Questionnaire
SI-KJ: Schlafinventar für Kinder und Jugendliche
EBI: Eltern-Belastungs-Inventar.

## Trial status

Protocol version number and date: 12.12.2024 version 3

The start date of the recruitment: 24.07.2023

Approximate date of recruitment completion: 31.12.2023
