# Supplementary material for: Promoting sleep and mental well-being in children: Protocol for a naturalistic pilot in-app study among users of the Aumio app
Source: PLoS One. 2025 Apr 29;20(4):e0322302. doi: 10.1371/journal.pone.0322302 (PMC12040105; doi:10.1371/journal.pone.0322302)
Supplement: S1 File — (DOCX) [file pone.0322302.s001.docx]

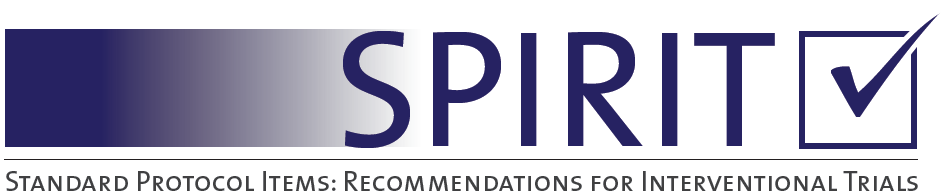


SPIRIT 2013 Checklist: Recommended items to address in a clinical trial protocol and related documents*

| Section/item | ItemNo | Description |
| --- | --- | --- |
| **Administrative information** | | |
| Title | 1 | Promoting sleep and mental well-being in children: A naturalistic pilot in-app study among users of the Aumio ® app |
| Trial registration | 2a | DRKS-ID of the study: DRKS00031147 |
|  | 2b | All items from the World Health Organization Trial Registration Data Set |
| Protocol version | 3 | 16.09.2023 version 1 |
| Funding | 4 | none |
| Roles and responsibilities | 5a | Freie Universität Berlin  Klinische Kinder- und Jugendpsychologie und -psychotherapie  Habelschwerdter Allee 45  14195 Berlin  Aumio GmbH  Firmensitz  Mühlenstraße 8a  14167 Berlin  Krankenhaus Porz am Rhein  Urbacher Weg 19  51145 Köln |
|  | 5b | Freie Universität Berlin  Prof. Claudia Calvano, PhD  Klinische Kinder- und Jugendpsychologie und -psychotherapie  Habelschwerdter Allee 45  14195 Berlin |
|  | 5c | In study design; collection, management, analysis, and interpretation of data; writing of the report; and the decision to submit the report for publication with authority over any these activities. |
|  | 5d | Not applicable. |
| Introduction |  |  |
| Background and rationale | 6a | The purpose of this study is to conduct an initial analysis of the usage behavior of the Aumio app and effectiveness on child's sleep quality. Further, an email psychoeducational module will be tested for its feasibility and initial effectiveness on parental stress and possibly child’s sleep problems as well. |
|  | 6b | Sleep problems are widespread among children in Germany. Simultaneously, sleep plays a crucial role in child development and child’s sleep problems can also negatively affect parents' sleep, health, and daytime functioning. |
| Objectives | 7 | 1) children’s sleep quality will be significantly increased and 2) health-related quality of life will be significantly. Furthermore, we hypothesize that parenting stress will be significantly reduced 12 weeks after the start of the use of the email psychoeducational program. |
| Trial design | 8 | The present research project is designed as a one-group pre-post pilot study. |
| Methods: Participants, interventions, and outcomes | | |
| Study setting | 9 | The study is a single study conducted exclusively in-app and within a German-speaking population. |
| Eligibility criteria | 10 | Participants are eligible if the children are between 6;0 and 12;0 years old. The following exclusion criteria will be collected, assessed, and applied at T0: (1) insufficient German reading and understanding language proficiency of the child or guardian, (2) impaired hearing ability of the child, (3) diagnosed intelligence impairment or other severe developmental disability of the child, and (4) diagnosed obstructive sleep apnea of the child. |
| Interventions | 11a | The participants will be provided access to the Aumio app and receive the parent-centred email psychoeducation module at weekly intervals. The intervention duration is set at twelve weeks. |
|  | 11b | Participants can discontinue their participating at any time by uninstalling the app from their mobile devices and opting out of the email list. The intervention does not require contact with the study team |
|  | 11c | No reminders of app use will be used. |
|  | 11d | non |
| Outcomes | 12 | For the assessment of child’s sleep quality, we used the Children's Sleep Habits Questionnaireand the Sleep inventory for children. We used the KiddyKINDL and KidKINDL to assess the health-related quality of life and the Parenting Stress Index to asses parenting stress. |
| Participant timeline | 13 | A total of three surveys will be conducted: at the beginning of the intervention and before the first app use (T0), six weeks after the first app use (T1), and immediately following the conclusion of the 12-week intervention (T2). The same measurement instruments will be used in each survey. |
| Sample size | 14 | A final required sample size of 456 is needed. |
| Recruitment | 15 | Study participants are mainly recruited through the Aumio app. Recruitment will be augmented by advertisements online and in pediatric clinics, where a link to the website in the survey platform for informed consent is provided. |
| **Methods: Assignment of interventions (for controlled trials)** | | |
| Allocation: |  |  |
| Sequence generation | 16a | Not applicable. |
| Allocation concealment mechanism | 16b | Not applicable. |
| Implementation | 16c | Not applicable. |
| Blinding (masking) | 17a | Not applicable. |
|  | 17b | Not applicable. |
| **Methods: Data collection, management, and analysis** | | |
| Data collection methods | 18a | The collection of the questionnaire data will be done using the German server of Tivian GmbH. |
|  | 18b | None. |
| Data management | 19 | The data of the questionnaires and the data of the app are collected separately, and the assignment is only possible via an assignment list, which are exclusively encrypted and password-protected and stored locally in separate folders. |
| Statistical methods | 20a | Descriptive methods and specification of missing observations are used to present socio demographic data, satisfaction and data on app use. Intervention effects on the outcomes are analyzed by repeated measures ANOVA. |
|  | 20b | No interim analyses on treatment effectiveness will be conducted. |
|  | 20c | Subgroup analyses will be conducted to examine whether effects differ between groups with different sociodemographic characteristics, e. g. household size, parental education, parental income, relationship conflict between parents, parental separation, co-sleeping and media use. |
| **Methods: Monitoring** | | |
| Data monitoring | 21a | The data of the questionnaires and the data of the app are collected separately, and the assignment is only possible via an assignment list, which are exclusively encrypted and password-protected and stored locally in separate folders. The hard drive will be located at Freie Universität Berlin. |
|  | 21b | No interim analyses on treatment effectiveness will be conducted. |
| Harms | 22 | We expect no adverse events, as the interventions and assessment tools imply neither burden nor risks. However, in case of spontaneously reported adverse events like increased psychological burden, the families can contact the study team at any time and in case any adverse event is reported, it will be followed up and necessary steps will be taken. |
| Auditing | 23 | No audits are planned. |
| Ethics and dissemination | | |
| Research ethics approval | 24 | Ethical approval and modification approval were granted by the Ethics Committee of the Department of Education and Psychology of Freie Universität Berlin on February 07, 2023, under reference number 047.2022. |
| Protocol amendments | 25 | Any deviations from or changes to the protocol will be documented. Significant changes will be reported to the Ethics Committee of the Freie Universität Berlin and documented in the German Clinical Trials Registry. |
| Consent or assent | 26a | Informed consent will be obtained from all guardians as well as children in child-friendly language prior to screening at T0. Only individuals who provide informed consent and child assent can participate in the study. |
|  | 26b | Not applicable. |
| Confidentiality | 27 | The data of the questionnaires and the data of the app are collected separately, and the assignment is only possible via an assignment list, which are exclusively encrypted and password-protected and stored locally in separate folders. The hard drive will be located at Freie Universität Berlin. |
| Declaration of interests | 28 | NR, CC and AW declare they have no competing interest for the overall trial and the results. JO is the founder of the Aumio app. MR is staff at Aumio and conceptualised the parent module. JO provided access to the app, enabling in-app recruitment. JO and MR are not involved in the evaluation (data analysis, interpretation and writing). |
| Access to data | 29 | This is a protocol paper, and no data is available yet. |
| Ancillary and post-trial care | 30 | We expect no adverse events, as the interventions and assessment tools imply neither burden nor risks. However, in case of spontaneously reported adverse events like increased psychological burden, the families can contact the study team at any time and in case any adverse event is reported, it will be followed up and necessary steps will be taken. |
| Dissemination policy | 31a | The results of the study will be submitted for publication in relevant journals and on the Aumio website. |
|  | 31b | All authors contributed to the design of the study protocol. CC and NKR wrote the first version of the protocol. All authors reviewed the paper, provided input, and approved the final version of the paper. |
|  | 31c | This is a protocol paper, and no data is available yet. |
| Appendices |  |  |
| Informed consent materials | 32 | Consent form and other related documentation given to participants were reviewed by the ethics Ethics Committee of the Freie Universität Berlin |
| Biological specimens | 33 | Not applicable. |

*It is strongly recommended that this checklist be read in conjunction with the SPIRIT 2013 Explanation & Elaboration for important clarification on the items. Amendments to the protocol should be tracked and dated. The SPIRIT checklist is copyrighted by the SPIRIT Group under the Creative Commons “[Attribution-NonCommercial-NoDerivs 3.0 Unported](http://www.creativecommons.org/licenses/by-nc-nd/3.0/)” license.
