# Supplementary material for: Promoting sleep and mental well-being in children: Protocol for a naturalistic pilot in-app study among users of the Aumio app
Source: PLoS One. 2025 Apr 29;20(4):e0322302. doi: 10.1371/journal.pone.0322302 (PMC12040105; doi:10.1371/journal.pone.0322302)
Supplement: S2 File — (DOCX) [file pone.0322302.s002.docx]

# Application for advice from the Ethics Committee on the implementation of a scientific project

#

# 20.01.2023

#

1. **title of the study/research project**

Aumio - a mobile-based app for the promotion of sleep and mental well-being for children: a pilot study

## Applicants / Study leaders

**(names, affiliation, address, e-mail addresses)**

Prof. Dr. Claudia Calvano, Klinische Kinder- und Jugendpsychologie und -psychotherapie, Habelschwerdter Allee 45, 14195 Berlin, claudia.calvano@fu-berlin.de

Nadja Kristin Ruckser, Studentin im Masterstudiengang Psychologie mit Schwerpunkt Klinische Psychologie und Psychotherapie, Fachbereich Erziehungswissenschaften und Psychologie, Freie Universität Berlin, Habelschwerdter Allee 45, 14195 Berlin, E-Mail: nadja.ruckser@fu-berlin.de

1. **cooperation partners**

(names, affiliation)

Aumio GmbH, represented by Jean Ochel (co-founder)

1. **type of study/research project**

**☒** Scientific research

**☒** Bachelor/Master thesis/PhD

supervised by: Prof. Dr. Claudia Calvano

☐ Scientific internship

☐ other:

## general conditions of the study/research project

The project is based on an application for funding (e.g. from a third-party funding body)

☐ Yes

founded by:

Application enclosed:

☐ Yes

☐ No

☐ will be submitted later

☒ No

An opinion of the ethics committee was issued by a third party (e.g. third-party funder).

☒ required

☐ not required

## Ethik

The research project described in this application has already been reviewed by an ethics committee of another institution.

☐ Yes, with a positive vote (Please enclose original application and ethics vote!)

☐ Yes, with a negative vote (Please enclose original application and ethics vote!)

☒ No

The research project described in this application is very similar in terms of research question, methods and test subjects to a research project that has already been assessed by the Ethics Committee of the Department of Education and Psychology at the FU Berlin.

☒ Yes (Please enclose original application and ethics vote!)

☐ No

## quick checkup

**YES NO**

All test subjects have full legal capacity

Vulnerable subjects (groups) will participate (e.g. patients,

persons with disabilities or learning disabilities, etc.).

There is a deception about the content, purpose, method, setting and participation.

Questions are asked that are of an intimate nature or whose answers

answering them can be perceived as stigmatizing.

There may be indications of suicidal tendencies.

Incidental findings may occur.

There are physical or mental burdens and/or risks for the subjects.

test persons.

The test subjects are given medication, placebos or

or other substances.

Blood and/or tissue samples are taken.

The EU data protection guidelines are complied with.

1. **short description of the research project (max. 300 words)**

(Theoretical background, aim and hypotheses)

Sleep is of great importance for child development and is closely linked to physical, cognitive, behavioral and socio-emotional functions that affect children's learning, behavior and general well-being (Schliebera & Han, 2021). However, in preschool and primary school age, sleep problems and a variety of associated impairments can occur (Armstrong et al., 2014). Problems with falling asleep or staying asleep are reported particularly frequently (Schwerdtle et al., 2016). The effects of children's sleep problems range from daytime sleepiness, impaired everyday functioning and a short attention span (Schliebera & Han, 2021) to negative consequences for learning, memory and academic performance (Dewald et al., 2010). In addition, sleep problems are correlated with negative behavioural reactions and interactions with peers, ADHD and a more frequent occurrence of externalizing behaviours (Schliebera & Han, 2021). The inclusion of parents in children's sleep training is particularly relevant not only due to the fact that childhood sleep problems can also have a negative impact on parents' sleep, health and functioning (Meltzer & Westin, 2011), but also because parents can influence children's therapy processes and outcomes (Kölch et al., 2015). In einer Meta-Analyse von Zhu, Xiao und Tu (2022) wurden Hinweise dafür gefunden, dass technologiebasierte Interventionen zu einer Verbesserung des kindlichen Schlafs beitragen können. Zudem haben digitale Gesundheitsanwendungen (E-Mental Health) das Potential, bedarfsorientiert eingesetzt zu werden (Dockweiler & Fischer, 2019). A meta-analysis by Zhu, Xiao and Tu (2022) found evidence that technology-based interventions can contribute to improving children's sleep. In addition, digital health applications (e-mental health) have the potential to be used in a demand-oriented manner (Dockweiler & Fischer, 2019).

**Aim**

The aim of this pilot study is to test the feasibility of the Aumio app and the parent-centered module as the basis for a future clinical study. The aim is to test whether its use improves children's sleep. In addition, effects on the health-related quality of life and psychological problem areas of children are tested. The effects of using the app on parental stress are also examined.

**Hypotheses**

1. the children show a greater improvement in sleep after using the Aumio app and the parent-centered module compared to before use (primary endpoint).

2. the children show a greater improvement in health-related quality of life after using the Aumio app and the parent-centered module compared to before use (secondary endpoint)

3. the children show a greater reduction in mental health problems after using the Aumio app and the parent-centered module compared to before use (secondary endpoint)

4. the children's parents show a greater reduction in parental stress after using the Aumio app and the parent-centered module compared to before use (secondary endpoint).

## Method

(Procedure and design of the study, description of the tasks, survey methods and questionnaires used, description of any follow-up studies requested)

**Study design**

The present research project is a one-group pre-post design. The participants will be given access to the Aumio app and will also be sent a parent-centered module at regular intervals. The duration of the intervention is six-week periods. Surveys will take place at T0, T1 (six-weeks after T0) and T2 (three-months after T0).

**The Aumio App**

Aumio is a smartphone app to promote healthy sleep and strengthen children's mental health. The app was developed as a scientific project as part of a research project at the Free University of Berlin. Aumio is now designed by clinical psychologists with the co-creative support of children, guardians and experts. The app focuses on children's sleep as well as relaxation, concentration and emotions. In the sleep module, Aumio offers a variety of audio stories, fantasy journeys and sleep sounds to make going to bed as relaxing as possible for children. The Aumio app also includes psychoeducation and playful learning of relaxation techniques using meditation and dream journeys. The meditation techniques are taught in a child-friendly way and made easily accessible for children. The exercises and stories are based on techniques such as autogenic training or progressive muscle relaxation. A special focus of the psychoeducation is the involvement of parents and guardians by also giving them tips for putting their child to sleep. The Aumio app gives families low-threshold access to measures for their children's mental health. The meditations and dream journeys for falling asleep last between 5 and 20 minutes. In the intervention, families are given the choice of when and how often Aumio should be used in the evening. At the beginning of the intervention, the parents/guardians are given guidelines from other users whose children use the Aumio app 2-3 times a week and listen to 1-2 dream journeys or meditations each time. In addition, the parents/guardians in the intervention group are sent the parent-centered module by email several times a week. This provides them with psychoeducational knowledge on the topic of children's sleep.

**Study program**

The study participants are recruited in the Aumio app and then directed to a special page in the app. There they (children and legal guardians) are informed about the purpose and procedure of the study as well as the possible advantages and disadvantages of participation. They are informed that participation in the study is completely voluntary and that participation can be terminated at any time without giving reasons. In addition, all participants will be informed about their rights with regard to data storage, data protection and data deletion. Before the screening at T0, the declaration of consent is obtained from both the legal guardians and the children in child-friendly language in the app. After agreeing to the conditions of participation, the legal guardians are forwarded to the first part of our study (T0). There, the initial screening of the parents and children begins, in which sociodemographic characteristics (e.g. age of the child and parents, type of school attended by the child) and relevant covariates, which were identified in advance in a literature review, are collected. The relevant covariates can be divided into family characteristics and characteristics of the mother, father and child. The family characteristics are household size, parental education (Hale, Berger, LeBourgeois, & Brooks-Gunn, 2009), parental income (McDowall, Elder, & Campbell, 2017), relationship conflict between parents (El-Sheikh, Hinnant, & Erath, 2015), parental separation (Rudd, Holtzworth-Munroe, D'Onofrio, & Waldron, 2019) and co-sleeping (Peng, Yuan, & Ma, 2019). The characteristics of the child are media consumption before bedtime and the presence of media in the sleep environment (Falbe, Davison, Franckle, Ganter, Gortmaker, Smith, Land, & Taveras, 2015; Van den Bulck, 2004). The outcomes are then assessed using parent versions.

The primary outcome sleep is assessed using the German version of the Children Sleep Habits Questionnaire (CSHQ-DE; 45 items) by Schlarb (2016). This instrument is a screening questionnaire about the sleep of children aged 4 to 10 years filled out by their parents or guardians. It was validated and standardized by Schlarb, Schwerdtle and Hautzinger (2010) on a German standard sample of 4-10-year-old children. The Screening Instrument of the Sleep Inventory in parents' external report (SI-KJ; Lehmkuhl, Agache, Alfer, Fricke-Oerkermann, Tielsch, Mitschke, Schäfermeier, van der Stouwe, & Wiater, 2015; 33 items) serves as an additional source of information. This instrument has been validated and standardized on German norm samples of 5-11-year-old children and parents.

The following secondary outcomes are surveyed: The children's health-related quality of life using the parent version of the KiddyKINDL or the KidKINDL (Ravens-Sieberer & Bullinger, 1998a; Ravens-Sieberer & Bullinger, 1998b; 24 items). The KiddyKINDL is administered to parents/guardians of 4-6 year old children and the KidKINDL to parents/guardians of 7-12 year old children. Both versions have been psychometrically tested (e.g. Ellert, Ravens-Sieberer, Erhart, et al., 2011) and a German norm sample is available (Ravens-Sieberer, Ellert & Erhart, 2007). The German version of the Strengths and Difficulties Questionnaire (SDQ; Goodman, 1997; 25 items) by Klasen, Woerner, Rothenberger and Goodman (2003) was selected to assess psychological problems. The instrument is a behavioural screening for 2-17-year-old children for which validation and standardization studies are available (Klasen, Woerner, Rothenberger, & Goodman, 2003). Parental stress is assessed using the subscales on the impairment of parental functional areas of the Parental Stress Inventory (EBI; Tröster, 2011). Feasiblity is to be assessed based on the number of users, dropout rates and usage behavior.

After completing the screening, participants are provided with the Aumio app free of charge. In addition, the parent-centered module is sent to them by email several times a week at regular intervals. The duration of the intervention is set at six weeks. Participants can decide for themselves how they use the app in the evening. At the beginning of the intervention, the parents are given guidelines from other users. They typically use the Aumio app two to three times a week and listen to one or two dream journeys or meditations each time. The meditations and dream journeys have a total playing time of between 5 and 20 minutes. At the end of the six weeks, the second measurement time point (T1) takes place. The same instruments as at T0 are collected here. In addition, two items are used to determine whether the parents or guardians in the intervention group have read the parent-centered module and integrated it into their everyday lives~~.~~ The third measurement point (T2) is three months after the start of the intervention. At this point, the same instruments as at T0 are collected again.

The intention-to-treat principle is used in the main analysis. The socio-demographic characteristics and other relevant covariates of the participants are summarized descriptively, including missing observations. The intervention effects on the primary and secondary outcomes are tested using T-tests for dependent samples. In addition, the average use of the app is examined to determine the effects on sleep. Dose response analyses will be used to determine when the parents or children have used the app sufficiently. In addition, a regression of sleep on the initial CSHQ score and the number of times the app was used was calculated.

## Sample

(Recruitment of test subjects, description of sample and sample size, inclusion and exclusion criteria, compensation, handling of missing or limited business and/or decision-making capacity, e.g. in children)

The study participants are recruited in the Aumio app. For participation in the study, free access to the full range of the Aumio app will be provided. Potential participants will be included if they fulfill the following inclusion criteria: (1) age of the child: 4;0 to 12;0 years, (2) willingness and ability (e.g. sufficient knowledge of German, sufficient skills in using the smartphone of the child and the parent/guardian). The following exclusion criteria are collected, evaluated and applied at T0: (1) poor German language skills of the child or legal guardian, (2) impaired hearing, (3). The legal guardians must agree to a declaration of consent for the child to participate in the study. The child will also be informed about the study and the child's consent is required.

1. **Physical, mental or emotional strain on the participants**

(fatigue, exertion, invasive procedures, medication, drug tests, aversive stimuli, negative experiences; resulting risks for participants)

The study includes the use of the Aumio app. In addition, only online questionnaires are collected. The app is designed in such a way that its use increases the well-being of the participants. However, there is a potential risk that using the app could lead to fatigue or be perceived as an effort. In addition, paying attention to one's own inner states can certainly give rise to negative feelings. The children and their legal guardians are therefore informed in advance that the exercises can be interrupted at any time and continued at a later time (Appendix A: Participant information with declaration of consent). Answering the questionnaires can also be tiring. The answers are therefore provided by the parents or guardians. In addition, both using the app and completing the questionnaires take time. This can be perceived as aversive, which is why information about the exact course of the study, including the number of measurement times, duration of the mindfulness exercises, etc., is provided in the participant information. In general, however, the risks are considered to be low, as all the points mentioned are voluntary and can be terminated at any time without negative consequences.

1. **Information, consent, deception (unless evident from the annexes)** (voluntariness, consent, possibility of withdrawal, complete vs. incomplete information, deception, information if necessary, for intervention studies: references to control conditions and allocation to conditions)

Participation in the study is voluntary. The legal guardians of the participating children must give their consent. The children will also be informed about the study in a child-friendly manner and must give their consent to participate. Participants are informed that they have the right to discontinue the study at any time without giving reasons.

1. **Data protection (unless evident from the annexes)**

Information on the collection, storage, further processing and deletion of data; disclosure of data to third parties, including open data; publication of group-related results; information on personal data; pseudonymization/anonymization; right to deletion of data; information on confidentiality and data secrecy)

See appendix on data protection

1. **Dealing with abnormal findings (optional)**

(e.g. in the case of EEG, MRI or diagnostic tests; including neurological abnormalities, mental disorders, suicidal tendencies; if there are indications of suicidal tendencies, please describe the procedure for dealing with them)

Only screening instruments that do not allow diagnoses are used. For this reason, no diagnoses are reported back to the participants.

1. **Bibliography**

Barkmann, C., Erhart, M., Schulte-Markwort, M., & BELLA Study Group (2008). The German version of the Centre for Epidemiological Studies Depression Scale for Children: Psychometric evaluation in a population-based survey of 7 to 17 years old children and adolescents - Results of the BELLA study. *European Child and Adolescent Psychiatry*, *17*, 116-124. https://doi.org/10.1007/s00787-008-1013-0

Birmaher, B., Brent, D. A., Chiappetta, L., Bridge, J., Monga, S., & Baugher, M. (1999). Psychometric properties of the Screen for Child Anxiety Related Emotional Disorders (SCARED): A replication study. *Journal of the American Academy of Child and Adolescent Psychiatry*, *38*(10), 1230–1236. https://doi.org/10.1097/00004583-199910000-00011

Dewald, J. F., Meijer, A. M., Oort, F. J., Kerkhof, G. A., & Bögels, S. M. (2010). The influence of sleep quality, sleep duration and sleepiness on school performance in children and adolescents: A meta-analytic review. *Sleep medicine reviews*, *14*(3), 179–189. https://doi.org/10.1016/j.smrv.2009.10.004

Dockweiler, C., & Fischer, F. (2019). Digitale Gesundheit: Eine Einführung. *Aphasie und verwandte Gebiete, 1*(45), 6-13. https://pub.uni-bielefeld.de/download/2935379/2935380/apha_fachzeitschrift_1_2019web.pdf

Döpfner, M., & Görtz-Dorten, A. (2017). *Diagnostik-System für psychische Störungen nach ICD-10 und DSM-5 für Kinder- und Jugendliche (DISYPS-III)*. Hogrefe.

Ravens-Sieberer, Ellert, U., & Erhart, M. (2007). Gesundheitsbezogene Lebensqualität von Kindern und Jugendlichen in Deutschland: eine Normstichprobe für Deutschland aus dem Kinder- und Jugendgesundheitssurvey (KiGGS). *Bundesgesundheitsblatt, Gesundheitsforschung, Gesundheitsschutz*, *50*(5/6), 810–818. https://doi.org/10.1007/s00103-007-0244-4Ellert, U., Ravens-Sieberer, U., Erhart, M. et al. (2011). Determinants of agreement between self-reported and parent-assessed quality of life for children in Germany - Results of the German Health Interview and Examination Survey for Children and Adolescents (KiGGS). *Health and Quality of Life Outcomes*, *9*(102). https://doi.org/10.1186/1477-7525-9-102

El-Sheikh, M., Hinnant, J. B., & Erath, S. A. (2015). Marital conflict, vagal regulation, and children’s sleep: A longitudinal investigation. *Monographs of the Society for Research in Child Development*, *80*(1), 89–106. https://doi.org/10.1111/mono.12146

Erhart, M., Ottova, V., Gaspar, T., Jericek, H., Schnohr, C., Alikasifoglu, M., Morgan, A., Ravens-Sieberer, U., & HBSC Positive Health Focus Group (2009). Measuring mental health and well-being of school-children in 15 European countries using the KIDSCREEN-10 Index. *International Journal of Public Health*, *54*(2), 160–166. https://doi.org/10.1007/s00038-009-5407-7

Falbe, F., Davison, K. K., Franckle, R. L., Ganter, C., Gortmaker, S. L., Smith, L., Land, T., & Taveras, E. M. (2015). Sleep Duration, Restfulness, and Screens in the Sleep Environment. *Pediatrics*, *135*(2), e367–e375. https://doi.org/10.1542/peds.2014-2306

Goodman R (1997) The Strengths and Difficulties Questionnaire: A research note. *Journal of Child Psychology and Psychiatry*, *38*, 581-586.

Hale, L., Berger, L. M., LeBourgeois, M. K., & Brooks-Gunn, J. (2009). Social and demographic predictors of preschoolers' bedtime routines. *Journal of Developmental and Behavioral Pediatrics*, *30*(5), 394-402. https://doi.org/10.1097/DBP.0b013e3181ba0e64

Kölch, M., Dockhorn, M., Moser, I., & Fegert, J.M. (2015). Einbezug von Eltern in der Kinder- und Jugendpsychiatrie. In Aktion Psychisch Kranke, P. Weiß & A. Heinz (Eds.), *Qualität therapeutischer Beziehung* (pp. 108-115). Aktion Psychisch Kranke.

Lehmkuhl, G., Agache, A., Alfer, D., Fricke-Oerkermann, L., Tielsch, C., Mitschke, A., Schäfermeier, E., van der Stouwe, J., & Wiater, A. (2015). *Schlafinventar für Kinder und Jugendliche SI-KJ*. Hogrefe.

Schlieber, M., & Han, J. (2021). The role of sleep in young children’s development: A review. *The Journal of Genetic Psychology*, *182*(4), 205-217, DOI: 10.1080/00221325.2021.1908218

Mattejat, F., & Remschmidt, H. (1999). *Fragebögen zur Beurteilung der Behandlung (FBB)*. Hogrefe.

McDowall, P. S., Elder, D. E., & Campbell, A.J. (2017). Relationship between parent knowledge of child sleep, and child sleep practices and problems: A pilot study in a children's hospital cohort. *Journal of Pediatrics and Child Health*, *53*(8), 788-793. https://doi.org/10.1111/jpc.13542.

Meltzer, L. J., & Westin, A. M. L. (2011). Impact of child sleep disturbances on parent sleep and daytime functioning. In M. El-Sheikh (Ed.), *Sleep and development: Familial and socio-cultural considerations* (pp. 113–131). Oxford University Press. https://doi.org/10.1093/acprof:oso/9780195395754.003.0006

Peng, X., Yuan, G., & Ma, N. (2019). Cosleeping and sleep problems in children: A systematic review and meta-analysis. *Sleep and Biological Rhythms*, *17*(4), 367–378. https://doi.org/10.1007/s41105-019-00226-z

Ravens-Sieberer, U. & Bullinger, M. (1998a). Assessing health related quality of life in chronically ill children with the German KINDL: first psychometric and content-analytical results. *Quality of Life Research*, *7*(5), 399-407.

Ravens-Sieberer, U. & Bullinger, M. (1998b). News from the KINDL-Questionnaire – A new version for adolescents. Quality of Life Research, *7*, 653.

Rudd, B. N., Holtzworth-Munroe, M., D’Onofrio, B. M., & Waldron, M. (2019). Parental relationship dissolution and child development: the role of child sleep quality. *Sleep*, *42*(2), 1–10.https://doi.org/10.1093/sleep/zsy224

Schlarb, A. (2016). Fragebogen zu kindlichen Schlafgewohnheiten Children Sleep Habits Questionnaire (CSHQ-DE). In H. Schulz, P. Geisler, A. Rodenbeck & Deutsche Gesellschaft für Schlafforschung und Schlafmedizin (Eds.), *Kompendium Schlafmedizin* (p. 1). ecomed.

Schlarb, A., Schwerdtle, B. & Hautzinger, M. (2010). Validation and psychometric properties of the German version of the Children’s Sleep Habits Questionnaire (CSHQ-DE). *Somnologie,* *14,* 260–266. https://doi.org/10.1007/s11818-010-0495-4

Schwerdtle, B., Roeser, K., Kübler, & Schlarb, A. A. (2010). Validierung und psychometrische Eigenschaften der deutschen Version des Sleep Self Report (SSR-DE). *Somnologie*, *14*, 267- 274. https://doi.org/10.1007/s11818-010-0496-3

Tröster, H. (2011). *Eltern-Belastungs-Inventar: EBI; deutsche Version des Parenting Stress Index (PSI) von RR Abidin*. Hogrefe.

Van den Bulck, J. (2004). Television viewing, computer game playing, and Internet use and self-reported time to bed and time out of bed in secondary-school children. *Sleep*, *27*, 101–104. https://doi.org/10.1093/sleep/27.1.101

Zhu, H., Xiao, L., & Tu, A. (2022). Effectiveness of technology-based interventions for improving sleep among children: A systematic review and meta-analysis, *Sleep Medicine*, *91*, 141-150.https://doi.org/10.1016/j.sleep.2022.02.013

## Additional information for the ethics committee (optional)

## Appendix

☒ A) Participant information with declaration of consent

☒ B) Data protection information

☒ C) Emails to the participants of the study

☒ D) Previous ethics application with ethics vote

☒ I am familiar with the regulations for the ethical evaluation of research projects of the Department of Education and Psychology at Freie Universität Berlin.

_____________________________ ___________________________________

Place, date Signature

_______________________________ ___________________________________

Place, date Signature

_______________________________ ____________________________________

Place, date Signature of supervisor (thesis)

##

1. **Participant information with declaration of consent**

**Initial information on participation in the study (displayed before part 1 of T0 at Unipark)**

Thank you for your interest in participating in the "Aumio" online study. We would like to inform you about some important points before you give your consent to participate in the study. Please read the information carefully and feel free to ask any questions you may have using the contact details below.

**What is the background to the study?**

The initial findings from the research suggest that various mindfulness-based exercises conducted over several weeks may have a positive impact on children's sleep and related factors. The "Aumio" app was developed to guide and accompany children in engaging with these exercises in a playful space-themed setting. In the Sleep module, "Aumio" offers a variety of audio stories, imaginative journeys, and soothing sounds to create a relaxing bedtime routine for children. Each exercise lasts between five and twenty minutes and is child-friendly. Additionally, "Aumio" has created a module for parents, providing information about children's sleep and offering practical sleep tips. In our study, we aim to investigate whether the use of the app and the parent-centered module successfully improves children's sleep. We will also assess potential effects of using "Aumio" on children's health-related quality of life, mental health, and parental stress.

**Who can participate in the study?**

The study is intended for children between 4 and 12 years of age. Additionally, your child should be able to understand spoken German for participation and should not have impaired hearing. Additionally, both you and your child should have sufficient competence in using a smartphone.

**What happens now?**

After the agreement to the participation conditions by the legal guardians and the children, initially, one of the legal guardians of the child should fill out the first questionnaire for participation in the study. The same person should also complete the subsequent questionnaires that we will send to you in the future. In the end, we would also kindly ask you to provide an email address where we can reach you. We will use your email address to send you the parent-centered module and then, as the study progresses, to send you the additional questionnaires and reminders for participation.

**How will it procced?**

Initially, you will receive further information about the study, along with a link to another questionnaire via email. Once you have filled out the questionnaire, you will receive a code from us that allows you to use the Aumio app free of charge. Six weeks and three months after receiving the app, we will send you another questionnaire each time. The provided version of the app can be used throughout the entire study period and beyond.

**What are the participation conditions?**

By participating in this study, you and your child make an important contribution to demonstrating the effectiveness of the "Aumio" app. Demographic variables, sleep, psychological issues and parental burden will be assessed. These data will be used in pseudonymous form for scientific research and publications. Further information about the handling of your data can be found in our "Data Protection Information." There is no financial compensation for participating in the study.

We would like to inform you that participation in the study is voluntary and can be terminated by you at any time without providing reasons, without any disadvantages for you or your child. We do not expect that completing the questionnaires or using "Aumio" will have any negative effects. Please remember that you and your child are not obligated to do anything that makes you feel uncomfortable. If you notice any negative effects of participating in the study, please contact us immediately. In emergencies, please contact your local emergency services.

In order for your child to participate in the study, all legal guardians must consent to the participation. So, if you have shared custody, the second legal guardian must also agree. If you have sole custody of your child, you are allowed to decide on your child's participation in the study alone. Your child is actively involved in the study, so it is important that your child also agrees to participate in the study.

**More questions and contact options?**

If you have any questions about the study, please feel free to contact Nadja Kristin Ruckser at any time at n.ruckser@fu-berlin.de.

**Responsibility**

The study is part of a master's thesis and is supervised by Prof. Dr. Claudia Calvano.

Habelschwerdter Allee 45

Room JK 24/121

14195 Berlin

Email: claudia.calvano@fu-berlin.de

Phone number: +49 30 838 585 70

1. **Digital Consent Form**

I have understood the information for participants as well as the data protection information. I am aware that participation is voluntary, and I or my child can withdraw from participation at any time without stating reasons, without any disadvantages to me or my child. I agree that my data and my child's data will be stored pseudonymized and used for scientific purposes. I hereby confirm that all legally authorized guardians of the child consent to participation.

Yes

No

Information for Your Child's Participation (will be displayed during Part 1 at T0 before the children's survey on Unipark)

Here are child-friendly explanations about the study for your child. It is important to us that your child understands what the study is about and also agrees to participate. If your child cannot read and understand all of this information alone, you are welcome to read the text to them and help them understand. However, the decision to participate should be made by your child alone.

Many children sometimes have difficulties with sleep. This can be very tough. That's why we want to help children who feel this way with the "Aumio" app. With the app, you do a few minutes of exercises. There is no "right" or "wrong" in these exercises. You should listen to a voice and try to think about different things that the voice tells you. In the future, we will also ask your parents a few times about how well you can sleep and how you are feeling. This will help us find out if we can help you and other children with "Aumio." Participating with us is completely voluntary. This means that if you no longer want to participate, you can stop at any time and do not have to do any more exercises or answer questions.

I would like to participate.

Yes

No

**Additional Information for Study Participants about the "Aumio" App (will be sent to participants by email after T0)**

With this email, we are sending you the code that allows you to use the "Aumio" app for free. "Aumio" provides mindfulness exercises in a space-themed playful setting. Information on how to install the app is included in the email. The app explains how to use it, and it is designed for your child to use regularly. Typically, users of "Aumio" use the app two to three times a week and listen to one to two dream journeys each time. Six weeks after sending the app, we will send you another link to the online questionnaires. After another three months, we will send you a final link to the online questionnaires.

**How do the exercises in Aumio work?**

In the Sleep module, Aumio offers a variety of bedtime stories, imaginary journeys, and soothing sounds to make children's bedtime as relaxing as possible. Additionally, the Aumio app includes psychoeducation and playful learning of relaxation techniques through meditations and dream journeys. The meditation techniques are presented in a child-friendly manner and made easily accessible for children. The exercises and stories are based on techniques such as autogenic training or progressive muscle relaxation. A special focus of psychoeducation is involving the parents by providing them with bedtime tips for the child. During the exercises, your child can practice, under guidance, focusing on their own breath, for example. The audio recordings provide precise instructions on how the exercises should be performed. By following the instructions with good intentions, there is no "wrong" way to do these exercises, as they are different for everyone. The exercises can be done either sitting or lying down. When choosing between sitting and lying down, please let your child decide. If they choose to sit, they should sit upright to ensure proper breathing. Each session lasts approximately five to twenty minutes.

**Can I participate?**

Of course, you are also invited to participate in the exercises. It's best to discuss with your child beforehand if it feels right for them. We even believe that it could be beneficial if you set a good example by participating in the exercises together. This way, your child will realize that these exercises are a good thing. Additionally, sharing this experience together might potentially enhance the effects of the program.

**How and when can you best perform the exercises?**

Your child is best advised to carry out the exercises in a quiet place, such as their bedroom with the door closed, so that no one can disturb or make noise during the minutes of the exercise. The location should ideally remain consistent throughout the course so that a routine can be established. This routine helps in better execution of the exercises.

Commonly chosen times, especially in the Sleep module, are in the evenings before bedtime. Simply consider when it works best for you and your child, taking your child's preferences into account. Like the location, it's also advantageous to keep the timing consistent throughout the course, which aids in the exercise's regularity.

**How regularly should the exercises be performed?**

The course is designed to allow you and your child to decide when and how often you want to do an exercise. Typically, users of "Aumio" use the app two to three times a week and listen to one or two dream journeys each time. However, there may be days when you don't have time for an exercise or you forget to do it. This won't have any disadvantages for you or your child.

In general, we recommend that you approach the exercises without pressure so that your child can develop their own interest and enjoyment in the exercises. In other words, if it doesn't fit into the day or feels forced, it's better to skip it and plan it for the next day.

**What to do if your child doesn't feel like it?**

It's important that your child consents to the exercise. It's perfectly normal for your child to not feel like doing the exercises at times. In such cases, you can tell your child that you understand it can be tiring or even boring to do the exercises. But these exercises can help them have more joy and fun in other life situations. Invite your child to give it another try while observing their own boredom or effort as if they were observing their breath. Your child can also explore where the feeling is located in their body and try to accept that it's there. Of course, in such a situation, it can also help if you lead by example and do the exercise yourself. However, if your child doesn't feel like it on a particular day, it shouldn't lead to a conflict, and the exercise can be postponed to the following day.

**What are the participation conditions?**

By participating in this study, you and your child make an important contribution to demonstrating the effectiveness of the "Aumio" app. This involves collecting demographic variables, sleep data, psychological problem areas and parental stress. These data will be used in pseudonymous form for scientific work and publications. Further information on how we handle your data can be found in our "Data Protection Information." There is no financial compensation for participating in the study. We want to emphasize that participation in the study is voluntary and can be discontinued at any time without stating reasons, without any disadvantages for you or your child. We do not expect that filling out the questionnaires or using "Aumio" will have any negative effects. Please remember that you and your child are not obliged to do anything that makes you uncomfortable. If you notice any negative effects of participating in the study, please contact us immediately. In case of emergencies, please contact your local emergency service. In order for your child to participate in the study, all legal guardians must agree to the participation. So, if you have joint custody, the second legal guardian must also agree. If you have sole custody of your child, you can decide alone about your child's participation in the study. Since your child is actively involved in the study, it's important that your child also agrees to participate.

**What's the next steps?**

With this email, you will receive the installation file for the "Aumio" app. Six weeks after that, we will send you another set of online questionnaires. Six weeks later, we will send you a final online survey.

**More questions and contact options?**

If you have any questions about the study, feel free to contact Nadja Kristin Ruckser anytime at n.ruckser@fu-berlin.de.

**Responsibility**

The study is part of a master's thesis and is supervised by Prof. Dr. Claudia Calvano

Habelschwerdter Allee 45

Room JK 24/121

14195 Berlin

Email: claudia.calvano@fu-berlin.de

Phone number: +49 30 838 585 70

1. **Data Protection Information**

**General Data Protection Information:**

The protection of your data is very important to us, which is why all collected information is treated strictly confidential and in accordance with legal requirements. The study serves exclusively scientific purposes. During the study, personal information about your child's sleep and related psychological issues and parental stress will be collected. This data will be collected through various questionnaires via "Unipark." Additional data regarding the use of the "Aumio" app (e.g., how many sessions per week were completed) will be collected through the app itself. The data from the questionnaires and the data from the app are collected separately. The link between the data in the app and the data from the Unipark surveys can only be established through an allocation list, which is stored exclusively on encrypted and password-protected USB drives. The data on the USB drives is encrypted, and the USB drives themselves are password-protected. The USB drives and the external hard drive are stored in a locked cabinet at the Freie Universität Berlin. The following sections describe the data protection measures for the separate data collection locations.

**Data Collection:**

**a) Questionnaires via Unipark**

The link to the survey in Unipark will be sent to you by email. In the course of this survey, personal and mental health data about you and your child will be provided. To enable the (pseudo-)anonymous collection, an identifier/code is included in the email, which participants should enter at the beginning of the Unipark survey. The questionnaires are provided via a German server operated by Questback GmbH (UniPark). This company specializes in secure data collection for research purposes and is ISO 27001 certified by the Federal Office for Information Security. The transmission of respondents' answers to the questionnaire platform is done with SSL encryption. Additionally, unauthorized individuals do not have access to the data processing systems at any time and, therefore, cannot view, read, copy, alter, or remove the data.

**b) Data Collection via the "Aumio App"**

The "Aumio" app does not collect sensitive, personally identifiable data. Only data with technical background (such as device identification and information, language, operating version, etc.) and user behavior data are collected, such as how many mindfulness sessions were completed or how long the app was used. Furthermore, all this data in the "Aumio" app is only collected under a pseudonym, which participants received in a separate email.

**Data Storage:**

**a) Questionnaires via Unipark**

Access to the answered questionnaires, which include data about your child's sleep, associated psychological issues your stress, and socio-economic data, is only possible through password protection on the Questback GmbH platform. Only authorized personnel, i.e., the study team, have password-protected access. After the data collection is completed, the data will be deleted from the platform and stored exclusively locally on the study team's computers, as well as on an external hard drive (as a backup). The answers from the questionnaires do not contain real names and can only be attributed through an identifier. The allocation list, containing the real names or email addresses of the participants and their corresponding identifiers, is accessible only to the study team, encrypted and password-protected, stored locally on a separate USB drive. The allocation list is thus kept separately from the data, ensuring that unauthorized individuals cannot associate the data with real names or email addresses.

**b) Data Collection via the "Aumio" App**

The data collected through the "Aumio" app is only accessible to the administrators of Aumio and is provided to the study team by them. Aumio UG (haftungsbeschränkt) also operates under all applicable data security and data protection laws, including the EU's applicable data protection regulations. The transfer of this data also takes place through a password-protected USB drive, where the data is again encrypted.

**Evaluation:**

The allocation of data from Unipark and data from the "Aumio" app is done through an allocation list. This list is password-protected, encrypted, and only accessible to the study team. The acquired data is merged and then evaluated in an anonymized form. No conclusions about individual persons are possible. The data is only evaluated as a whole, not in smaller individual groups, so no conclusions can be drawn in this way either. In this form, the data is also only accessible to the study team. All data is stored locally or on a hard drive (as a backup) with password protection. The required password is only accessible to the study team. The study team is bound by confidentiality regarding personal data and commits to comply with the Federal Data Protection Act. No personal information will be disclosed or published to third parties.

According to the General Data Protection Regulation (GDPR), you have the following rights:

• Information about the processing of personal data (Art. 15)

• Revocation of consent given (Art. 7)

• Correction (Art. 16)

• Deletion (Art. 17)

• Restriction of processing (Art. 18)

• Right to complain to a supervisory authority (Art. 77)

Exercising any of the above rights will not have any negative consequences for you.

Deletion of Data:

According to the GDPR, you have the right to request the deletion of your data at any time. Otherwise, the retention period for anonymized data is 10 years. Personal data will be deleted immediately as soon as it is no longer necessary for the conduct of the study (i.e., after the completion of the study

). Specifically for our study, we will store your personal data only as long as you participate in the study and beyond that until the data can be evaluated within the scope of our research questions. Accordingly, we set ourselves a storage period of one year. Afterward, your data will be deleted by us.

You can contact the responsible data protection officer of Freie Universität Berlin, Dr. Karsten Kinast, at: datenschutz@fu-berlin.de

## E-Mails to the participants during the study

1. Feedback/Invitations via Unipark: Feedback after completing the 1st survey (T0)

Subject: Aumio Study: Confirmation of Survey Completion

Dear participant,

Thank you for completing the questionnaires. We will now evaluate your responses and provide you with feedback on the further progress of our study in the coming days.

Best regards,

Nadja Kristin Ruckser

1. Feedback for not meeting the study's inclusion criteria

Subject: Aumio Study: Feedback on Study Inclusion/Exclusion

Dear participant,

We regret to inform you that the results of the questionnaires do not meet the inclusion criteria of our study. This does not mean that your distress or needs are not significant. Therefore, we would recommend the following links if you would like to explore the topic further:

Information on childhood sleep problems

https://www.dgkj.de/eltern/dgkj-elterninformationen/elterninfo-kind-schlaeft-nicht

https://psychologische-coronahilfe.de/beitrag/schlafstoerungen-bei-kindern-und-jugendlichen/

https://www.kindergesundheit-info.de/themen/schlafen/schlafprobleme/unterstuetzung-und-hilfe/

https://www.kindergesundheit-info.de/themen/schlafen/schlafprobleme/haeufige-probleme/

Find help:

https://www.psychenet.de/de/hilfe-finden/schnelle-hilfe/krisenanlaufstellen-kj.html

If you have any questions, please feel free to contact us at the email address n.ruckser@fu-berlin.de.

Best regards,

Nadja Kristin Ruckser

1. Feedback upon meeting the study's inclusion criteria

Subject: Aumio Study: Feedback on Study Inclusion/Exclusion - Invitation to Survey

Dear participant,

We are pleased to inform you that the results of the questionnaires meet the inclusion criteria of our study, and we are including your child and you in our study. The next step is to complete another online questionnaire. You can find it at the following link:

***www.unipark.xyw/123***

In this survey, you are requested to answer the questions on your own, without your child. This will take approximately xx minutes. After completing the questionnaire, you will receive another email from us, informing you whether you belong to the intervention group and will receive the "Aumio" app immediately or whether you belong to the waiting group and will receive the app after six weeks.

If you have any questions, please do not hesitate to contact us at the provided email address.

Best regards,

Nadja Kristin Ruckser

n.ruckser@fu-berlin.de

1. Feedback after completing the 2nd survey (T0, Part 2),

Subject: Aumio Study: Code for Free Use of the Aumio App

Dear participant,

Thank you for completing the second survey! You and your child are now part of our study.

Attached, you will find an installation file for the app along with instructions on how to install it.

To sign up for the app, you will need your participant ID, which you created in Unipark. Here's a reminder of the format you used to create it:

Xx from xx

Xx from xx

Xx xx

For free access to the Aumio app, please enter the following code in the app: Xx xx

In the participant information, you will find instructions on how to use the app. In summary, use "Aumio" in a way that fits best into your child's and your daily life. Ideally, engage in the exercises several times a week, but if that doesn't work out, it's not a problem. You are welcome to join and participate in the exercises if your child wishes.

In six weeks, you will receive another link from us leading to another survey. It will include the same questionnaires you encountered in the first and second surveys, and, as before, we will only be directing questions to you.

If you have any questions, please feel free to reach out to us.

Best regards,

Nadja Kristin Ruckser

1. Invitation to T1 (Both Groups)

Subject: Aumio Study: Invitation to Survey

Dear participant,

We would like to invite you to participate in our survey once again. It will include the same questionnaires you have already encountered. As in our initial survey, we will only be directing questions to you, and this part will take approximately xx minutes.

Below is the link to the survey:

***www.unipark.xyw/123***

If you have any questions, please feel free to reach out to us!

Best regards,

Nadja Kristin Ruckser

1. Feedback after Completing T1,

Subject: Confirmation of Survey Completion

Dear participant,

Thank you for completing the survey again. You will receive another invitation to participate in another survey from us in six weeks. This survey will include the same questionnaires you have already encountered. As in our initial survey, we will only be directing questions to you. In the meantime, feel free to continue using "Aumio" as you wish.

If you have any questions, please feel free to reach out to us!

Best regards,

Nadja Kristin Ruckser

1. Invitation to T2 (Both Groups)

Subject: Aumio Study: Invitation to the Final Survey

Dear participant,

It's that time again: we would like to invite you to complete our survey once more. The procedure remains the same: we will exclusively ask questions to you. This part will take approximately xx minutes. Below, you will find the link to the survey:

***www.unipark.xyw/123***

If you have any questions, please feel free to reach out to us!

Best regards,

Nadja Kristin Ruckser

1. Feedback after Completing T2 (Both Groups)

Subject: End of the Study

Dear participant,

Thank you for completing the third survey! We are delighted that you have stayed with us for so long. Even though the study is now officially over for you, you are welcome to continue using "Aumio." The app will remain available to you.

We wish you all the best!

Best regards,

Nadja Kristin Ruckser

1. Reminder T1/T2 (to remind via Gmail)

Subject: Reminder to Complete 2nd/3rd Survey

Dear participant,

First of all, we would like to thank you for being a part of our study!

Recently, we sent you a link to the second/third survey. If you have already completed it, you can ignore this email. However, if you haven't filled out the survey yet, we would like to remind you to please do so.

We understand that you are likely very busy, but from a study perspective, it is essential that you take approximately XX minutes to complete the second/third part of the study. Without your input at this stage, we wouldn't be able to analyze the data effectively. Only with the new data can we determine the impact of the Aumio app.

If you have any questions, please feel free to reach out to me at n.ruckser@fu-berlin.de.

Thank you in advance!

Best regards,

Nadja Kristin Ruckser
