# Supplementary material for: Promoting sleep and mental well-being in children: Protocol for a naturalistic pilot in-app study among users of the Aumio app
Source: PLoS One. 2025 Apr 29;20(4):e0322302. doi: 10.1371/journal.pone.0322302 (PMC12040105; doi:10.1371/journal.pone.0322302)
Supplement: S3 File — (DOCX) [file pone.0322302.s003.docx]

# Antrag auf Beratung durch die Ethikkommission zur Durchführung eines wissenschaftlichen Vorhabens

#

# 20.01.2023

#

## Titel der Studie/des Forschungsvorhabens

Aumio - a mobile-based app for the promotion of sleep and mental well-being for children: a pilot study

## AntragstellerInnen / LeiterInnen der Studie

(Namen, Affiliation, Anschrift, E-Mail-Adressen)

Prof. Dr. Claudia Calvano, Klinische Kinder- und Jugendpsychologie und -psychotherapie, Habelschwerdter Allee 45, 14195 Berlin, claudia.calvano@fu-berlin.de

Nadja Kristin Ruckser, Studentin im Masterstudiengang Psychologie mit Schwerpunkt Klinische Psychologie und Psychotherapie, Fachbereich Erziehungswissenschaften und Psychologie, Freie Universität Berlin, Habelschwerdter Allee 45, 14195 Berlin, E-Mail: nadja.ruckser@fu-berlin.de

## KooperationspartnerInnen

(Namen, Affiliation)

Aumio GmbH, vertreten durch Jean Ochel (Mitgründer)

## Art der Studie/des Forschungsvorhabens

**☒** Wissenschaftliche Forschung

**☒** Bachelor-/Masterarbeit/Promotion

betreut durch: Prof. Dr. Claudia Calvano

☐ Wissenschaftliches Praktikum

☐ Sonstiges:

## Rahmenbedingungen der Studie/des Forschungsvorhabens

Dem Vorhaben liegt ein Antrag auf Finanzierung (z.B. durch einen Drittmittelgeber) zugrunde

☐ Ja

gefördert durch:

Antrag liegt bei: ☐ Ja

☐ Nein

☐ wird nachgereicht

☒ Nein

Eine Stellungnahme der Ethikkommission wurde von dritter Stelle (z.B. Drittmittelgeber)

☒ verlangt

☐ nicht verlangt

## Anzeige

Das in diesem Antrag beschriebene Forschungsvorhaben wurde bereits durch eine Ethikkommission einer anderen Institution begutachtet.

☐ Ja, mit positivem Votum (Bitte Originalantrag und Ethikvotum beifügen!)

☐ Ja, mit negativem Votum (Bitte Originalantrag und Ethikvotum beifügen!)

☒ Nein

Das in diesem Antrag beschriebene Forschungsvorhaben weist in Bezug auf Fragestellung, Methoden und Probanden eine hohe Ähnlichkeit mit einem Forschungsprojekt auf, welches schon durch die Ethikkommission des FB Erziehungswissenschaft und Psychologie der FU Berlin begutachtet wurde.

☒ Ja (Bitte Originalantrag und Ethikvotum beifügen!)

☐ Nein

## Kurz-Checkup

**JA NEIN**

Alle Probanden sind voll geschäftsfähig.

Es werden vulnerable Probanden(-gruppen) teilnehmen (z.B. Patienten,

Personen mit Behinderung oder Lernstörung etc.).

Es liegt eine Täuschung über Inhalt, Zweck, Methode, Setting und die

Teilnahme vor.

Es werden Fragen gestellt, die intimer Natur sind oder deren

Beantwortung als stigmatisierend wahrgenommen werden kann.

Es können Hinweise auf Suizidalität auftreten.

Es können Zufallsbefunde auftreten.

Es gibt körperliche oder mentale Belastungen und/oder Risiken für die

Probanden.

Den Probanden werden Medikamente, Placebos oder

andere Substanzen verabreicht.

Es werden Blut- und/oder Gewebeproben entnommen.

Die EU-Datenschutzrichtlinien werden eingehalten.

## Kurzbeschreibung des Forschungsvorhabens (max. 300 Wörter)

(Theoretischer Hintergrund, Ziel und Hypothesen)

Schlaf hat eine große Bedeutung für die kindliche Entwicklung und ist eng verknüpft mit körperlichen, kognitiven, verhaltensbezogenen und sozio-emotionalen Funktionen, die sich auf das Lernen, das Verhalten und das allgemeine Wohlbefinden von Kindern auswirken (Schliebera & Han, 2021). Im Vor- und Grundschulalter können allerdings Schlafprobleme sowie eine Vielzahl an damit assoziierten Beeinträchtigen auftreten (Armstrong et al., 2014). Besonders häufig wird von Problemen mit dem Ein- oder Durchschlafen berichtet (Schwerdtle et al., 2016). Auswirkungen von kindlichen Schlafproblemen reichen über Tagesmüdigkeit, einer Beeinträchtigung des alltäglichen Funktionsniveaus, einer kurzen Aufmerksamkeitsspanne (Schliebera & Han, 2021) bis hin zu negativen Folgen auf das Lernen, das Gedächtnis und die schulischen Leistungen (Dewald et al., 2010). Zudem sind Schlafprobleme mit negativen Verhaltensreaktionen und Interaktionen mit Gleichaltrigen, ADHS und einem häufigeren Auftreten von externalisierenden Verhaltensweisen korreliert (Schliebera & Han, 2021). Nicht nur aufgrund der Tatsache, dass sich kindliche Schlafprobleme auch negativ auf den Schlaf, die Gesundheit und das Funktionieren der Eltern auswirken können (Meltzer & Westin, 2011), ist der Einbezug der Eltern in das Einschlaftraining der Kinder besonders relevant, sondern auch weil die Eltern die Therapieprozesse und outcomes der Kinder beeinflussen können (Kölch et al., 2015). In einer Meta-Analyse von Zhu, Xiao und Tu (2022) wurden Hinweise dafür gefunden, dass technologiebasierte Interventionen zu einer Verbesserung des kindlichen Schlafs beitragen können. Zudem haben digitale Gesundheitsanwendungen (E-Mental Health) das Potential, bedarfsorientiert eingesetzt zu werden (Dockweiler & Fischer, 2019).

**Ziel**

Ziel dieser Pilotstudie ist die Überprüfung der Machbarkeit der Aumio App und des elternzentrierten Moduls als Grundlage für eine künftige klinische Studie. Dafür soll geprüft werden, ob deren Nutzung den Schlaf von Kindern verbessert. Zudem sollen Effekte auf die gesundheitsbezogene Lebensqualität und psychische Problembereiche von Kindern geprüft werden. Zusätzlich wird geprüft, welche Effekte die Nutzung der App auf die elterliche Belastung hat.

**Hypothesen**

1. Die Kinder zeigen nach der Nutzung der Aumio App und des elternzentrierten Moduls eine stärkere Verbesserung des Schlafes im Vergleich zu vor der Nutzung (primärer Endpunkt).
2. Die Kinder zeigen nach der Nutzung der Aumio App und des elternzentrierten Moduls eine stärkere Verbesserung der gesundheitsbezogenen Lebensqualität im Vergleich zu vor der Nutzung (sekundärer Endpunkt).
3. Die Kinder zeigen nach der Nutzung der Aumio App und des elternzentrierten Moduls eine stärkere Reduktion von seelischen Problemen im Vergleich zu vor der Nutzung (sekundärer Endpunkt).
4. Die Eltern der Kinder zeigen nach der Nutzung der Aumio App und des elternzentrierten Moduls eine stärkere Reduktion der elterlichen Belastung im Vergleich zu vor der Nutzung (sekundärer Endpunkt).

## Methode

(Ablauf und Design der Studie, Beschreibung der eingesetzten Aufgaben, Erhebungsmethoden und Fragebögen, ggf. Beschreibung von Folgestudien, die mitbeantragt werden)

**Studiendesign**

Das vorliegende Forschungsvorhaben ist als Ein-Gruppen Prä-Post-Design konzipiert. Den Teilnehmenden wird die Aumio App zugänglich gemacht und zusätzlich ein elternzentriertes Modul in regelmäßigen Abständen zugesandt. Die Dauer der Intervention umfasst zwölf Wochen. Erhebungen finden zu T0, T1 (sechs Wochen nach T0) und T2 (drei Monate nach T0) statt.

**Die Aumio App**

Aumio ist eine Smartphone App zur Förderung des gesunden Schlafs sowie zur Stärkung der psychischen Gesundheit von Kindern. Die App wurde als wissenschaftliches Projekt im Rahmen einer Forschungsarbeit an der Freien Universität Berlin entwickelt. Mittlerweile wird Aumio von klinischen Psycholog*innen unter ko-kreativer Unterstützung von Kindern, Erziehungsberechtigten und Expert*innen gestaltet. Die App widmet sich mit einem Fokus dem kindlichen Schlaf sowie Themen der Entspannung, Konzentration und Gefühlen. In dem Modul Schlaf bietet Aumio eine Vielzahl an Hörgeschichten, Fantasiereisen und Einschlafklängen um Kindern das abendliche Zubettgehen so entspannt wie möglich zu gestalten. Zudem ist in die Aumio App Psychoedukation und das spielerische Erlernen von Techniken zur Entspannung mittels Meditationen und Traumreisen integriert. Die Meditationstechniken werden kindgerecht vermittelt und für Kinder leicht zugänglich gemacht. Die Übungen und Geschichten beruhen auf Techniken wie dem autogenen Training oder der progressiver Muskelentspannung. Ein besonderer Fokus der Psychoedukation ist das Einbinden der Erziehungsberechtigten, indem auch ihnen Einschlaftipps für das Kind gegeben werden. Familien erhalten mit der Aumio App einen niedrigschwelligen Zugang zu Maßnahmen für die psychische Gesundheit ihrer Kinder. Die Meditationen und Traumreisen zum Einschlafen haben eine Spieldauer zwischen 5 und 20 Minuten. In der Intervention wird den Familien freigestellt, wann und wie häufig Aumio am Abend genutzt werden soll. Als Orientierung werden den Erziehungsberechtigten zu Beginn der Intervention Richtwerte von anderen Nutzer*innen mitgegeben, deren Kinder die Aumio App 2-3x pro Woche nutzen und dabei je 1-2 Traumreisen oder Meditationen hören. Zusätzlich wird den Erziehungsberechtigten der Interventionsgruppe mehrmals wöchentlich das elternzentrierte Modul per Mail zugeschickt. In diesem erhalten sie psychoedukatives Wissen zu dem Thema kindlicher Schlaf.

**Studienablauf**

Die Studienteilnehmenden werden in der Aumio App rekrutiert und anschließend auf eine spezielle Seite in der App geleitet. Dort werden sie (Kinder und Erziehungsberechtigte) über den Zweck und den Ablauf der Studie sowie über mögliche Vor- und Nachteile der Teilnahme informiert. Sie werden in Kenntnis gesetzt, dass die Teilnahme an der Studie vollkommen freiwillig ist und die Teilnahme jederzeit ohne Angabe von Gründen beendet werden kann. Zudem werden alle Teilnehmenden über ihre Rechte in Bezug auf die Datenspeicherung, den Datenschutz und die Datenlöschung informiert. Vor dem Screening zu T0 wird die Einverständniserklärung sowohl von den Erziehungsberechtigten als auch von den Kindern in kindgerechter Sprache in der App eingeholt. Nach dem Zustimmen zu den Teilnahmebedingungen werden die Erziehungsberechtigten zu dem ersten Teil unserer Studie (T0) weitergeleitet. Dort beginnt das anfängliche Screening der Erziehungsberechtigten und Kinder, bei dem soziodemografische Merkmale (z. B. Alter des Kindes und der Eltern, Besuchte Schulform des Kindes) und relevante Kovariaten, die in einer Literaturrecherche vorab identifiziert wurden, erhoben werden. Die relevanten Kovariaten können in familiäre Merkmale sowie Merkmale der Mutter, des Vaters und des Kindes unterschieden werden. Die familiären Merkmale sind die Haushaltsgröße, die Bildung der Eltern (Hale, Berger, LeBourgeois, & Brooks-Gunn, 2009), das Einkommen der Eltern (McDowall, Elder, & Campbell, 2017), Beziehungskonflikte zwischen den Eltern (El-Sheikh, Hinnant, & Erath, 2015), die Trennung der Eltern (Rudd, Holtzworth-Munroe, D’Onofrio, & Waldron, 2019) und das gemeinsame Schlafen in einem Bett (Peng, Yuan, & Ma, 2019). Die Merkmale des Kindes sind Medienkonsum vor dem Schlafengehen und das Vorhandensein von Medien in der Schlafumgebung (Falbe, Davison, Franckle, Ganter, Gortmaker, Smith, Land, & Taveras, 2015; Van den Bulck, 2004). Anschließend werden die outcomes mittels Elternversionen erhoben.

Das primary outcome Schlaf wird mittels der deutschen Version des Children Sleep Habits Questionnaire (CSHQ-DE; 45 Items) von Schlarb (2016) erhoben. Dieses Instrument ist ein von den Erziehungsberechtigten ausgefüllter Screening-Fragebogen über den Schlaf von Kindern im Alter von 4 bis 10 Jahren. Es wurde von Schlarb, Schwerdtle und Hautzinger (2010) an einer deutschen Normstichprobe von 4-10-jährigen Kindern validiert und normiert. Als zusätzliche Informationsquelle dient das Screening Instrument des Schlafinventars im Fremdbericht der Eltern (SI-KJ; Lehmkuhl, Agache, Alfer, Fricke-Oerkermann, Tielsch, Mitschke, Schäfermeier, van der Stouwe, & Wiater, 2015; 33 Items). Dieses Instrument ist an deutschen Normstichproben von 5–11-jährigen Kindern und Eltern validiert und normiert worden.

Folgende secondary outcomes werden erhoben: Die gesundheitsbezogene Lebensqualität der Kinder mittels der Elternversion des KiddyKINDL beziehungsweise des KidKINDL (Ravens-Sieberer & Bullinger, 1998a; Ravens-Sieberer & Bullinger, 1998b; 24 Items). Der KiddyKINDL wird den Erziehungsberechtigten der 4-6-jährigen Kindern und der KidKINDL den Erziehungsberechtigten der 7-12-jährigen Kindern vorgelegt. Beide Versionen wurden psychometrisch geprüft (z. B. Ellert, Ravens-Sieberer, Erhart, et al., 2011) und es liegt eine deutsche Normstichprobe vor (Ravens-Sieberer, Ellert & Erhart, 2007). Zur Erfassung von psychischen Problemen wurde der Fragebogen zu Stärken und Schwächen (SDQ; Goodman, 1997; 25 Items) in der deutschen Version von Klasen, Woerner, Rothenberger und Goodman (2003) ausgewählt. Das Instrument ist ein Verhaltensscreening für 2-17-jährige Kinder, für das Validierungs- und Normierungsstudien vorliegen (Klasen, Woerner, Rothenberger, & Goodman, 2003). Die elterliche Belastung wird mittels der Subskalen über die Beeinträchtigungen elterlicher Funktionsbereiche des Eltern-Belastungs-Inventar (EBI; Tröster, 2011) erhoben. Die Feasiblity soll anhand der Anzahl der Nutzer*innen, der Abbruchraten und des Nutzungsverhaltens beurteilt werden.

Nach Durchführung des Screenings wird den Teilnehmenden die Aumio App kostenlos zur Verfügung gestellt. Zudem wird ihnen in regelmäßigen Abständen mehrmals wöchentlich das elternzentrierte Modul per Mail zugesandt. Die Dauer der Intervention ist auf zwölf Wochen festgelegt. Die Teilnehmenden können selbst entscheiden, wie sie die App am Abend nutzen. Zu Beginn der Intervention werden den Erziehungsberechtigten Richtwerte von anderen Nutzerinnen mitgegeben. Diese nutzen die Aumio App typischerweise zwei bis drei mal pro Woche und hören dabei je ein bis zwei Traumreisen oder Meditationen. Insgesamt haben die Meditationen und Traumreisen eine Spieldauer zwischen 5 und 20 Minuten. Nach Ablauf der sechs Wochen findet der zweite Messzeitpunkt (T1) statt. In diesem werden die gleichen Instrumente wie zu T0 erhoben. Zudem wird anhand von zwei Items abgefragt, ob die Erziehungsberechtigten der Interventionsgruppe das elternzentrierte Modul gelesen und in den Alltag integriert haben. Drei Monate nach Beginn der Intervention ist der dritte Messzeitpunkt (T2). In diesem werden erneut die gleichen Instrumente wie bei T0 erhoben.

In der Hauptanalyse wird das Intention-To-Treat Prinzip verwendet. Die soziodemografischen Merkmale und weiteren relevanten Kovariaten der Teilnehmenden werden deskriptiv inklusive der Angabe fehlender Beobachtungen zusammengefasst. Die Interventionseffekte auf die primary und secondary outcomes werden mittels T-tests für abhängige Stichproben geprüft. Zudem geprüft wird, was die durchschnittliche Nutzung der App sein muss, damit Effekte auf den Schlaf eintreten. Wann die Erziehungsberechtigen bzw. die Kinder die App ausreichend genutzt haben, wird mithilfe von Dose Response Analysen geprüft. Zudem wird eine Regression von dem Schlaf durch den initialen CSHQ Score & die Anzahl der Nutzungen der App berechnet.

## Stichprobe

(Rekrutierung der Versuchspersonen, Beschreibung von Stichprobe und Stichprobenumfang, Einschluss- und Ausschlusskriterien, Vergütung, Umgang bei fehlender oder eingeschränkter Geschäfts- und/oder Entscheidungsfähigkeit, z.B. bei Kindern)

Die Teilnehmenden werden in der Aumio App rekrutiert. Für die Teilnahme an der Studie wird ein kostenloser Zugang zu dem gesamten Angebot der Aumio App bereitgestellt. Die potenziellen Teilnehmenden werden eingeschlossen, wenn sie folgende Einschlusskriterien erfüllen: (1) Alter des Kindes: 4;0 bis 12 Jahre, (2) Bereitschaft und Fähigkeit (z. B. ausreichende Deutschkenntnisse, ausreichende Kompetenzen im Umgang mit dem Smartphone des Kindes und der Erziehungsberechtigten). Die folgenden Ausschlusskriterien werden bei T0 erhoben, bewertet und angewendet: (1) geringe Deutschkenntnisse des Kindes oder der Erziehungsberechtigten, (2) eine beeinträchtigte Hörfähigkeit, (3). Die Erziehungsberechtigten müssen einer Einverständniserklärung zustimmen, dass das Kind an der Studie teilnehmen darf. Das Kind wird ebenfalls über die Studie aufgeklärt und die Zustimmung des Kindes ist erforderlich.

## Körperliche, mentale oder emotionale Beanspruchung der Teilnehmenden

(Ermüdung, Anstrengung, invasive Verfahren, Medikamente, Arzneimitteltests, aversive Reize, negative Erfahrungen; daraus resultierende Risiken für Teilnehmende)

Die Studie beinhaltet die Nutzung der Aumio. Zudem werden ausschließlich Online-Fragebögen erhoben. Die App ist so gestaltet, dass ihre Verwendung das Wohlbefinden der Teilnehmenden steigert. Ein mögliches Risiko kann dennoch sein, dass die Verwendung zu Ermüdung führt oder als Anstrengung wahrgenommen wird. Zusätzlich kann das Achten auf die eigenen inneren Zustände durchaus negative Gefühle aufkommen lassen. Die Kinder und deren Erziehungsberechtigte werden daher vorab darauf hingewiesen, dass die Übungen jederzeit unterbrochen und zu einem späteren Zeitpunkt weitergeführt werden können (Anhang A: Teilnehmendeninformationen mit Einverständniserklärung). Auch die Beantwortung der Fragebögen kann als ermüdend erlebt werden. Die Beantwortung erfolgt daher von den Erziehungsberechtigten. Zusätzlich beanspruchen sowohl die App-Nutzung als auch das Ausfüllen der Fragebögen Zeit. Dies kann als aversiv empfunden werden, weshalb über den genauen Ablauf der Studie, inkl. Anzahl der Messzeitpunkte, Dauer der Achtsamkeitsübungen etc. in der Teilnehmdeninformation informiert wird. Generell sind die Risiken jedoch als klein einzuschätzen, da alle genannten Punkte freiwillig sind und jederzeit ohne negative Konsequenzen beendet werden können.

## Informiertheit, Einwilligung, Täuschung (soweit nicht aus den Anlagen ersichtlich)

(Freiwilligkeit, Einwilligung, Rücktrittsmöglichkeit, vollständige vs. unvollständige Informiertheit, Täuschung, ggf. Aufklärung, bei Interventionsstudien: Hinweise auf Kontrollbedingungen und Zuteilung zu Bedingungen)

Die Teilnahme an der Studie ist freiwillig. Die Erziehungsberechtigten der teilnehmenden Kinder müssen eine Einverständniserklärung geben. Die Kinder werden ebenfalls kindgerecht über die Studie informiert und müssen ihr Einverständnis zur Teilnahme geben. Die Teilnehmenden werden darüber informiert, dass sie jederzeit das Recht haben, die Studie ohne Angabe von Gründen abzubrechen.

## Datenschutz (soweit nicht aus den Anlagen ersichtlich)

(Angaben zu Erhebung, Speicherung, Weiterverarbeitung und Löschung von Daten; Weitergabe von Daten an Dritte, einschl. Open Data; Veröffentlichung gruppenbezogener Ergebnisse; Hinweise zu personenbezogenen Daten; Pseudonymisierung/Anonymisierung; Recht auf Löschung der Daten; Hinweise zu Schweigepflicht und Datengeheimnis)

Siehe Anhang zum Datenschutz

## Umgang mit auffälligen Befunden (optional)

(z.B. bei EEG-, MRT- oder testdiagnostischen Untersuchungen; einschl. neurologischer Auffälligkeiten, psychischen Störungen, Suizidalität; bei Hinweisen auf Suizidalität bitte Prozedere beschreiben, wie damit umgegangen wird)

Es werden ausschließlich Screening-Instrumente verwendet, die keine Diagnosen erlauben. Aus diesem Grund werden den Teilnehmenden keine Diagnosen rückgemeldet.

## Literaturverzeichnis

Barkmann, C., Erhart, M., Schulte-Markwort, M., & BELLA Study Group (2008). The German version of the Centre for Epidemiological Studies Depression Scale for Children: Psychometric evaluation in a population-based survey of 7 to 17 years old children and adolescents - Results of the BELLA study. *European Child and Adolescent Psychiatry*, *17*, 116-124. https://doi.org/10.1007/s00787-008-1013-0

Birmaher, B., Brent, D. A., Chiappetta, L., Bridge, J., Monga, S., & Baugher, M. (1999). Psychometric properties of the Screen for Child Anxiety Related Emotional Disorders (SCARED): A replication study. *Journal of the American Academy of Child and Adolescent Psychiatry*, *38*(10), 1230–1236. https://doi.org/10.1097/00004583-199910000-00011

Dewald, J. F., Meijer, A. M., Oort, F. J., Kerkhof, G. A., & Bögels, S. M. (2010). The influence of sleep quality, sleep duration and sleepiness on school performance in children and adolescents: A meta-analytic review. *Sleep medicine reviews*, *14*(3), 179–189. https://doi.org/10.1016/j.smrv.2009.10.004

Dockweiler, C., & Fischer, F. (2019). Digitale Gesundheit: Eine Einführung. *Aphasie und verwandte Gebiete, 1*(45), 6-13. https://pub.uni-bielefeld.de/download/2935379/2935380/apha_fachzeitschrift_1_2019web.pdf

Döpfner, M., & Görtz-Dorten, A. (2017). *Diagnostik-System für psychische Störungen nach ICD-10 und DSM-5 für Kinder- und Jugendliche (DISYPS-III)*. Hogrefe.

Ravens-Sieberer, Ellert, U., & Erhart, M. (2007). Gesundheitsbezogene Lebensqualität von Kindern und Jugendlichen in Deutschland: eine Normstichprobe für Deutschland aus dem Kinder- und Jugendgesundheitssurvey (KiGGS). *Bundesgesundheitsblatt, Gesundheitsforschung, Gesundheitsschutz*, *50*(5/6), 810–818. https://doi.org/10.1007/s00103-007-0244-4Ellert, U., Ravens-Sieberer, U., Erhart, M. et al. (2011). Determinants of agreement between self-reported and parent-assessed quality of life for children in Germany - Results of the German Health Interview and Examination Survey for Children and Adolescents (KiGGS). *Health and Quality of Life Outcomes*, *9*(102). https://doi.org/10.1186/1477-7525-9-102

El-Sheikh, M., Hinnant, J. B., & Erath, S. A. (2015). Marital conflict, vagal regulation, and children’s sleep: A longitudinal investigation. *Monographs of the Society for Research in Child Development*, *80*(1), 89–106. https://doi.org/10.1111/mono.12146

Erhart, M., Ottova, V., Gaspar, T., Jericek, H., Schnohr, C., Alikasifoglu, M., Morgan, A., Ravens-Sieberer, U., & HBSC Positive Health Focus Group (2009). Measuring mental health and well-being of school-children in 15 European countries using the KIDSCREEN-10 Index. *International Journal of Public Health*, *54*(2), 160–166. https://doi.org/10.1007/s00038-009-5407-7

Falbe, F., Davison, K. K., Franckle, R. L., Ganter, C., Gortmaker, S. L., Smith, L., Land, T., & Taveras, E. M. (2015). Sleep Duration, Restfulness, and Screens in the Sleep Environment. *Pediatrics*, *135*(2), e367–e375. https://doi.org/10.1542/peds.2014-2306

Goodman R (1997) The Strengths and Difficulties Questionnaire: A research note. *Journal of Child Psychology and Psychiatry*, *38*, 581-586.

Hale, L., Berger, L. M., LeBourgeois, M. K., & Brooks-Gunn, J. (2009). Social and demographic predictors of preschoolers' bedtime routines. *Journal of Developmental and Behavioral Pediatrics*, *30*(5), 394-402. https://doi.org/10.1097/DBP.0b013e3181ba0e64

Kölch, M., Dockhorn, M., Moser, I., & Fegert, J.M. (2015). Einbezug von Eltern in der Kinder- und Jugendpsychiatrie. In Aktion Psychisch Kranke, P. Weiß & A. Heinz (Eds.), *Qualität therapeutischer Beziehung* (pp. 108-115). Aktion Psychisch Kranke.

Lehmkuhl, G., Agache, A., Alfer, D., Fricke-Oerkermann, L., Tielsch, C., Mitschke, A., Schäfermeier, E., van der Stouwe, J., & Wiater, A. (2015). *Schlafinventar für Kinder und Jugendliche SI-KJ*. Hogrefe.

Schlieber, M., & Han, J. (2021). The role of sleep in young children’s development: A review. *The Journal of Genetic Psychology*, *182*(4), 205-217, DOI: 10.1080/00221325.2021.1908218

Mattejat, F., & Remschmidt, H. (1999). *Fragebögen zur Beurteilung der Behandlung (FBB)*. Hogrefe.

McDowall, P. S., Elder, D. E., & Campbell, A.J. (2017). Relationship between parent knowledge of child sleep, and child sleep practices and problems: A pilot study in a children's hospital cohort. *Journal of Pediatrics and Child Health*, *53*(8), 788-793. https://doi.org/10.1111/jpc.13542.

Meltzer, L. J., & Westin, A. M. L. (2011). Impact of child sleep disturbances on parent sleep and daytime functioning. In M. El-Sheikh (Ed.), *Sleep and development: Familial and socio-cultural considerations* (pp. 113–131). Oxford University Press. https://doi.org/10.1093/acprof:oso/9780195395754.003.0006

Peng, X., Yuan, G., & Ma, N. (2019). Cosleeping and sleep problems in children: A systematic review and meta-analysis. *Sleep and Biological Rhythms*, *17*(4), 367–378. https://doi.org/10.1007/s41105-019-00226-z

Ravens-Sieberer, U. & Bullinger, M. (1998a). Assessing health related quality of life in chronically ill children with the German KINDL: first psychometric and content-analytical results. *Quality of Life Research*, *7*(5), 399-407.

Ravens-Sieberer, U. & Bullinger, M. (1998b). News from the KINDL-Questionnaire – A new version for adolescents. Quality of Life Research, *7*, 653.

Rudd, B. N., Holtzworth-Munroe, M., D’Onofrio, B. M., & Waldron, M. (2019). Parental relationship dissolution and child development: the role of child sleep quality. *Sleep*, *42*(2), 1–10.https://doi.org/10.1093/sleep/zsy224

Schlarb, A. (2016). Fragebogen zu kindlichen Schlafgewohnheiten Children Sleep Habits Questionnaire (CSHQ-DE). In H. Schulz, P. Geisler, A. Rodenbeck & Deutsche Gesellschaft für Schlafforschung und Schlafmedizin (Eds.), *Kompendium Schlafmedizin* (p. 1). ecomed.

Schlarb, A., Schwerdtle, B. & Hautzinger, M. (2010). Validation and psychometric properties of the German version of the Children’s Sleep Habits Questionnaire (CSHQ-DE). *Somnologie,* *14,* 260–266. https://doi.org/10.1007/s11818-010-0495-4

Schwerdtle, B., Roeser, K., Kübler, & Schlarb, A. A. (2010). Validierung und psychometrische Eigenschaften der deutschen Version des Sleep Self Report (SSR-DE). *Somnologie*, *14*, 267- 274. https://doi.org/10.1007/s11818-010-0496-3

Tröster, H. (2011). *Eltern-Belastungs-Inventar: EBI; deutsche Version des Parenting Stress Index (PSI) von RR Abidin*. Hogrefe.

Van den Bulck, J. (2004). Television viewing, computer game playing, and Internet use and self-reported time to bed and time out of bed in secondary-school children. *Sleep*, *27*, 101–104. https://doi.org/10.1093/sleep/27.1.101

Zhu, H., Xiao, L., & Tu, A. (2022). Effectiveness of technology-based interventions for improving sleep among children: A systematic review and meta-analysis, *Sleep Medicine*, *91*, 141-150.https://doi.org/10.1016/j.sleep.2022.02.013

## Zusätzliche Informationen für die Ethikkommission (optional)

## Anlagen

☒ A) Teilnehmendeninformation mit Einverständniserklärung

☒ B) Datenschutzinformation

☒ C) E-Mails an die Teilnehmenden der Studie

☒ D) Früherer Ethikantrag mit Ethikvotum

☒ Die Ordnung zur ethischen Begutachtung von Forschungsprojekten des Fachbereichs Erziehungswissenschaft und Psychologie der Freien Universität Berlin ist mir bekannt.

_____________________________ ___________________________________

Ort, Datum Unterschrift

_______________________________ ___________________________________

Ort, Datum Unterschrift

_______________________________ ____________________________________

Ort, Datum Unterschrift BetreuerIn (Abschlussarbeit)

##

## Teilnehmendeninformation mit Einverständniserklärung

# *Erste Information zur Teilnahme an der Studie (wird vor Teil 1 von T0 bei Unipark angezeigt)*

Vielen Dank, dass Sie Interesse haben, an der Online-Studie „Aumio“ teilzunehmen. Gerne möchten wir Sie vor der Einwilligung zur Teilnahme an der Studie noch über einige wichtige Punkte aufklären. Bitte lesen Sie die Informationen sorgfältig und stellen Sie bei Unklarheiten gerne Fragen über die unten genannte Kontaktmöglichkeit.

**Was ist der Hintergrund für die Studie?**

Erste Ergebnisse aus der Forschung weisen darauf hin, dass sich verschiedene Übungen mit Inhalten der Achtsamkeit über mehrere Wochen positiv auf den Schlaf von Kindern und damit verbundenen Faktoren auswirken kann. Die App „Aumio“ wurde entwickelt, um verschiedene Übungen bei Kindern spielerisch in einem Weltraumsetting anzuleiten und zu begleiten. Im Modul Schlaf umfasst „Aumio” dabei eine Vielzahl an Hörgeschichten, Fantasiereisen und Einschlafklängen um Kindern das abendliche Zubettgehen so entspannt wie möglich zu gestalten. Die einzelnen Übungen haben eine Spieldauer zwischen fünf und zwanzig Minuten und werden kindgerecht durch die App angeleitet. Zusätzlich hat „Aumio“ ein Modul für Eltern und Erziehungsberechtigte entwickelt, das über den kindlichen Schlaf aufklärt und praktische Tipps zum Schlafen gibt. In unserer Studie möchten wir nun herausfinden, ob die Nutzung der App und des elternzentrierten Moduls den Schlaf der Kinder erfolgreich verbessert. Außerdem erheben wir mögliche Auswirkungen der Nutzung von „Aumio“ auf die gesundheitsbezogene Lebensqualität, psychische Problembereiche und die elterliche Belastung.

**Wer kann an der Studie teilnehmen?**

Die Studie ist für Kinder zwischen 4 und 12 Jahren gedacht. Des Weiteren sollte Ihr Kind für die Teilnahme gesprochenes Deutsch verstehen können und keine beeinträchtigte Hörfähigkeit haben. Zudem sollten Sie und Ihr Kind über ausreichende Kompetenzen im Umgang mit dem Smartphone verfügen.

**Was passiert jetzt?**

Nach der Zustimmung zu den Teilnahmebedingungen durch die Erziehungsberechtigten und die Kinder soll nun zunächst ein*e Erziehungsberechtigte*r des Kindes den ersten Fragebogen zur Teilnahme an der Studie ausfüllen. Die gleiche Person soll auch die weiteren Fragebögen ausfüllen, die wir Ihnen in Zukunft zusenden. Am Ende würden wir Sie noch darum bitten, eine E-Mail-Adresse anzugeben, unter der wir Sie erreichen können. Ihre E-Mail-Adresse verwenden wir auch, um Ihnen das elternzentrierte Modul zukommen zu lassen und dann im weiteren Verlauf der Studie zur Zusendung der weiteren Fragebögen sowie Erinnerung an die Teilnahme.

**Wie geht es danach weiter?**

Zunächst erhalten Sie weitere Informationen zu der Studie sowie einen Link zu einem weiteren Fragebogen per Mail. Nachdem Sie den Fragebogen ausgefüllt haben erhalten Sie von uns einen Code mit dem Sie die Aumio App kostenfrei nutzen können. Sechs Wochen und drei Monate nach Erhalt der App senden wir Ihnen jeweils einen weiteren Fragebogen zu. Die zur Verfügung gestellte Version der App kann dabei die gesamte Studienlaufzeit und auch darüber hinaus genutzt werden.

**Wie sind die Teilnahmebedingungen?**

Mit der Teilnahme an dieser Studie leisten Sie und Ihr Kind einen wichtigen Beitrag zu dem Wirksamkeitsnachweis der App „Aumio“. Dabei werden demografische Variablen, der Schlaf, psychische Problembereiche und die elterliche Belastung erhoben. Diese Daten werden in pseudonymisierter Form für wissenschaftliche Arbeiten sowie Publikationen verwendet. Weitere Informationen zum Umgang mit Ihren Daten können Sie in unseren „Informationen zum Datenschutz“ finden. Eine finanzielle Entlohnung für die Teilnahme an der Studie wird nicht geleistet.

Wir möchten Sie darauf hinweisen, dass die Teilnahme an der Studie freiwillig ist und jederzeit ohne Angabe von Gründen von Ihrer Seite aus abgebrochen werden kann, ohne dass dabei Nachteile für Sie oder Ihr Kind entstehen. Wir erwarten nicht, dass das Ausfüllen der Fragebögen oder die Verwendung von „Aumio“ negative Auswirkungen haben wird. Bitte denken Sie daran, dass Ihr Kind und Sie nichts tun müssen, was Ihnen ein unangenehmes Gefühl bereitet. Wenn Sie irgendwelche negativen Auswirkungen der Studienteilnahme feststellen, wenden Sie sich bitte umgehend an uns. In Notfällen wenden Sie sich bitte direkt an Ihren örtlichen Notdienst.

Damit Ihr Kind an der Studie teilnehmen darf, müssen alle gesetzlichen Erziehungsberechtigten der Teilnahme zustimmen. Wenn Sie also ein geteiltes Sorgerecht haben, dann muss auch die zweite erziehungsberechtigte Person einverstanden sein. Wenn Sie das alleinige Sorgerecht für Ihr Kind haben, dann dürfen Sie allein über die Teilnahme Ihres Kindes an der Studie entscheiden. Bei der Studie ist Ihr Kind stark beteiligt. Deswegen ist es wichtig, dass Ihr Kind ebenfalls mit der Teilnahme an der Studie einverstanden ist.

**Weitere Fragen und Kontaktmöglichkeiten?**

Bei Rückfragen zu der Studie kontaktieren Sie gerne jederzeit Nadja Kristin Ruckser unter n.ruckser@fu-berlin.de

**Verantwortlichkeit**

Die Studie findet im Rahmen von einer Masterarbeit statt und wird betreut durch Prof. Dr. Claudia Calvano

Habelschwerdter Allee 45

Raum JK 24/121

14195 Berlin

E-Mail: claudia.calvano@fu-berlin.de

Telefonnummer: +49 30 838 585 70

*Digitale Einverständniserklärung*

Ich habe die Informationen für Teilnehmende sowie die Informationen zum Datenschutz verstanden. Mir ist bewusst, dass die Teilnahme freiwillig ist und die Teilnahme jederzeit ohne Angabe von Gründen abgebrochen werden kann, ohne dass mir oder meinem Kind dadurch Nachteile entstehen. Ich bin damit einverstanden, dass meine Daten sowie die Daten meines Kindes pseudonymisiert gespeichert und zu wissenschaftlichen Zwecken verwendet werden. Ich versichere hiermit, dass alle gesetzlich Erziehungsberechtigten des Kindes mit der Teilnahme einverstanden sind.

Ja

Nein

*Informationen zur Teilnahme für Ihr Kind (wird während Teil 1 von T0 vor der Befragung der Kinder bei Unipark angezeigt)*

*Hier sind kindgerechte Informationen zu der Studie für Ihr Kind. Es ist uns wichtig, dass Ihr Kind weiß, worum es bei der Studie geht und ebenfalls mit der Teilnahme einverstanden ist. Wenn Ihr Kind diese Informationen nicht alleine lesen und verstehen kann, können Sie ihm*ihr gerne den Text vorlesen und beim Verständnis helfen. Die Entscheidung teilzunehmen, soll Ihr Kind jedoch alleine treffen.*

Viele Kinder haben manchmal Schwierigkeiten mit dem Schlafen. Das kann sehr schwierig sein. Deswegen möchten wir Kindern, denen es so geht, mit der App „Aumio“ helfen. Mit der App macht man ein paar Minuten eine Übung. Bei den Übungen gibt es kein „richtig“ oder „falsch“. Du sollst dabei einer Stimme zuhören und versuchen über verschiedene Dinge nachzudenken, die die Stimme dir nennt. Wir werden deine Eltern in Zukunft außerdem ein paar Mal fragen, wie gut du schlafen kannst und wie es dir so geht. Das wird uns dabei helfen herauszufinden, ob wir dir und anderen Kindern mit „Aumio“ helfen können. Das Mitmachen bei uns ist vollkommen freiwillig. Das heißt, wenn du nicht mehr mitmachen möchtest, kannst du jederzeit abbrechen und musst keine weiteren Übungen machen oder Fragen beantworten.

Ich möchte gerne teilnehmen

Ja

Nein

*Weitere Information für Teilnehmer*innen der Studie zu der App „Aumio“ (wird per E-Mail nach T0 an Teilnehmer*innen versendet)*

Mit dieser E-Mail schicken wir Ihnen nun den Code zu, mit dem Sie die „Aumio“ App kostenfrei nutzen können. „Aumio“ vermittelt spielerisch Achtsamkeitsübungen in einem Weltraumsetting. Der E-Mail sind Informationen zur App-Installation beigefügt. In der App wird die Nutzung erklärt. Die App ist darauf ausgerichtet, dass Ihr Kind diese regelmäßig nutzt. Typischerweise verwenden die Nutzer*innen von „Aumio“ die App zwei bis dreimal pro Woche und hören je ein bis zwei Traumreisen. Sechs Wochen nach dem Zusenden der App schicken wir Ihnen erneut einen Link zu den Online-Fragebögen zu. Nach weiteren drei Monaten schicken wir Ihnen ein letztes Mal einen Link zu den Online-Fragebögen.

**Wie laufen die Übungen in Aumio ab?**

In dem Modul Schlaf bietet „Aumio“ eine Vielzahl an Hörgeschichten, Fantasiereisen und Einschlafklängen um Kindern das abendliche Zubettgehen so entspannt wie möglich zu gestalten. Zudem ist in die „Aumio“ App Psychoedukation und das spielerische Erlernen von Techniken zur Entspannung mittels Meditationen und Traumreisen integriert. Die Meditationstechniken werden kindgerecht vermittelt und für Kinder leicht zugänglich gemacht. Die Übungen und Geschichten beruhen auf Techniken wie dem autogenen Training oder der progressiver Muskelentspannung. Ein besonderer Fokus der Psychoedukation ist das Einbinden der Erziehungsberechtigten, indem auch ihnen Einschlaftipps für das Kind gegeben werden. Während der Übungen kann Ihr Kind unter Anleitung üben, sich z.B. ganz auf den eigenen Atem fokussieren. Die Tonaufnahmen geben genau vor, wie die Übung ablaufen sollte. Wenn man den Anweisungen mit gutem Vorsatz folgt, kann also nichts „falsch“ gemacht werden, denn diese Übungen sind für jede*n anders. Die Übungen werden wahlweise im Sitzen oder Liegen durchgeführt. Lassen Sie bei der Wahl zwischen Sitzen und Liegen gerne Ihr Kind selbst entscheiden. Wenn es sitzt, sollte es jedoch möglichst aufrecht sitzen, sodass es gut atmen kann. Eine Einheit dauert in etwa fünf bis zwanzig Minuten.

**Darf ich auch bei den Übungen teilnehmen?**

Natürlich sind Sie auch eingeladen, an den Übungen teilzunehmen. Sprechen Sie am besten vorher mit Ihrem Kind ab, ob sich das für Ihr Kind gut anfühlt. Wir glauben sogar, dass es von Vorteil sein könnte, wenn Sie bei den Übungen mit gutem Beispiel vorangehen und sie diese gemeinsam machen. So wird Ihr Kind merken, dass diese Übungen eine gute Sache sind. Außerdem kann das gemeinsame Erlebnis die Effekte des Kurses womöglich noch verstärken.

**Wo und wann kann man am besten die Übungen durchführen?**

Am besten führt Ihr Kind die Übungen an einem ruhigen Ort durch, bspw. im Kinderzimmer mit geschlossener Tür, sodass in den Minuten der Übung keiner stören oder lärmen kann. Der Ort sollte über den Kurs hinweg möglichst gleich bleiben, sodass sich eine Gewöhnung einstellen kann. Diese Gewöhnung hilft, um die Übungen besser durchführen zu können.

Oft gewählte Zeiten sind im Modul Schlaf vor allem abends vor dem Schlafengehen. Überlegen Sie einfach, wann Sie es am besten mit Ihrem Kind einrichten können und beziehen Sie dabei die Wünsche Ihres Kindes mit ein. Wie beim Ort ist es auch bei der Zeit von Vorteil, diese möglichst über den Kurs gleich zu halten. Dies hilft ebenfalls bei der Durchführung der Übungen.

**Wie regelmäßig sollen die Übungen durchgeführt werden?**

Der Kurs ist darauf ausgerichtet, dass Sie und Ihr Kind selbst entscheiden können, wann und wie häufig Sie eine Übung durchführen. In der Regel benutzen die Nutzer*innen von „Aumio“ die App zwei bis dreimal pro Woche und hören je ein bis zwei Traumreisen. Selbstverständlich kann es aber auch Tage geben, an denen man keine Zeit für eine Übung hat oder man vergessen hat, sie durchzuführen. Dadurch entstehen keine Nachteile für Sie oder Ihr Kind.

Grundsätzlich empfehlen wir, dass Sie das Durchführen der Übungen jedoch ohne Druck angehen, sodass Ihr Kind möglichst ein eigenes Interesse und Freude an den Übungen entwickeln kann. Sprich, sollte es mal nicht in den Tag passen oder nur „reingequetscht“ werden können, lassen Sie es lieber aus und planen es für den Folgetag.

**Was tun, wenn mein Kind gar keine Lust hat?**

Es ist selbstverständlich wichtig, dass Ihr Kind in die Übung einwilligt. Dabei ist es ganz normal, wenn Ihr Kind mal keine Lust auf die Übungen hat. In diesem Fall können Sie Ihrem Kind sagen, dass Sie verstehen können, dass es anstrengend oder auch langweilig sein kann, die Übungen durchzuführen. Doch diese Übungen können ihr*ihm helfen, auch in anderen Lebenssituationen mehr Freude und Spaß zu haben. Laden Sie Ihr Kind also dazu ein, es noch einmal zu probieren und dabei die eigene Langeweile oder die Anstrengung einfach als etwas zu beobachten, wie es sonst den Atem beobachtet. Dabei kann Ihr Kind auch erkunden, wo das Gefühl im Körper sitzt, und versuchen zu akzeptieren, dass es da ist. Natürlich kann es in so einer Situation auch helfen, wenn Sie mit gutem Beispiel vorangehen und die Übung selbst mitmachen. Wenn Ihr Kind jedoch an einem Tag keine Lust hat, dann soll dies nicht zu einem Konflikt führen und die Übung lieber auf den Folgetag verschoben werden.

**Wie sind die Teilnahmebedingungen?**

Mit der Teilnahme an dieser Studie leisten Sie und Ihr Kind einen wichtigen Beitrag zu dem Wirksamkeitsnachweis der App „Aumio“. Dabei werden demografische Variablen, der Schlaf, psychische Problembereiche und die elterliche Belastung erhoben. Diese Daten werden in pseudonymisierter Form für wissenschaftliche Arbeiten sowie Publikationen verwendet. Weitere Informationen zum Umgang mit Ihren Daten können Sie in unseren „Informationen zum Datenschutz“ finden. Eine finanzielle Entlohnung für die Teilnahme an der Studie wird nicht geleistet. Wir möchten Sie darauf hinweisen, dass die Teilnahme an der Studie freiwillig ist und jederzeit ohne Angabe von Gründen von Ihrer Seite aus abgebrochen werden kann, ohne dass dabei Nachteile für Sie oder Ihr Kind entstehen. Wir erwarten nicht, dass das Ausfüllen der Fragebögen oder die Verwendung von „Aumio“ negative Auswirkungen haben wird. Bitte denken Sie daran, dass Ihr Kind und Sie nichts tun müssen, was Ihnen ein unangenehmes Gefühl gibt. Wenn Sie irgendwelche negativen Auswirkungen der Studienteilnahme feststellen, wenden Sie sich bitte umgehend an uns. In Notfällen wenden Sie sich bitte direkt an Ihren örtlichen Notdienst. Damit Ihr Kind an der Studie teilnehmen darf, müssen alle gesetzlichen Erziehungsberechtigten der Teilnahme zustimmen. Wenn Sie also ein geteiltes Sorgerecht haben, dann muss auch die zweite erziehungsberechtigte Person einverstanden sein. Wenn Sie das alleinige Sorgerecht für Ihr Kind haben, dann dürfen Sie allein über die Teilnahme Ihres Kindes an der Studie entscheiden. Bei der Studie ist Ihr Kind stark beteiligt. Deswegen ist es wichtig, dass Ihr Kind ebenfalls mit der Teilnahme an der Studie einverstanden ist.

**Der weitere Ablauf?**

Mit dieser E-Mail erhalten Sie von uns die Installationsdatei für die App „Aumio“. Sechs Wochen danach senden wir Ihnen erneut Online-Fragebögen zu. Weitere sechs Wochen später senden wir Ihnen ein letztes Mal eine Online-Umfrage zu.

**Weitere Fragen und Kontaktmöglichkeiten?**

Bei Rückfragen zu der Studie kontaktieren Sie gerne jederzeit Nadja Kristin Ruckser unter n.ruckser@fu-berlin.de

**Verantwortlichkeit**

Die Studie findet im Rahmen von einer Masterarbeit statt und wird betreut durch Prof. Dr. Claudia Calvano

Habelschwerdter Allee 45

Raum JK 24/121

14195 Berlin

E-Mail: claudia.calvano@fu-berlin.de

Telefonnummer: +49 30 838 585 70

## Datenschutzinformation

**Allgemeines zum Datenschutz**

Der Schutz Ihrer Daten ist uns sehr wichtig, weshalb alle erhobenen Informationen selbstverständlich streng vertraulich und nach den gesetzlichen Anforderungen behandelt werden. Die Studie dient ausschließlich wissenschaftlichen Zwecken. Während der Studie werden persönliche Informationen über den Schlaf Ihrer Kinder und damit verbundenen psychischen Problembereichen. Zusätzlich werden wir Sie zu Ihrer Belastung befragen. Diese Erhebung wird durch verschiedene Fragebögen über „Unipark“ erfolgen. Weitere Daten bezüglich der Nutzung der „Aumio“ App (z.B. wie viele Einheiten pro Woche absolviert worden sind) werden über die App selber erhoben. Die Daten der Fragebögen und die Daten der App werden dementsprechend getrennt erhoben. Die Zuordnung zwischen den Daten in der App und den Daten aus den Unipark-Erhebungen ist nur über eine Zuweisungsliste möglich, welche ausschließlich verschlüsselt und passwortgesichert lokal auf separaten USB-Sticks gespeichert sind. Die Daten auf den USB-Sticks werden verschlüsselt, die USB-Sticks selber werden passwortgeschützt sein. Die USB-Sticks und die externe Festplatte werden in einem verschlossenen Schrank an der Freien Universität Berlin aufbewahrt. Im Folgenden wird dargestellt, welche Datenschutzvorkehrungen für die getrennten Generierungsorte vorgesehen sind.

**Erhebung der Daten**

a) Fragebögen über Unipark

Der Link zu der Umfrage in Unipark wird Ihnen per E-Mail zugesendet. Im Rahmen dieser Erhebung werden Angaben zu personenbezogenen und psychischen Gesundheitsdaten von Ihnen und Ihrem Kind gemacht. Damit die Erhebung (pseudo-)anonymisiert erfolgen kann, ist in der E-Mail eine Kennziffer/ein Code enthalten, die die Teilnehmenden am Anfang der Umfrage in Unipark eingeben sollen. Die Fragebögen werden über einen deutschen Server der Questback GmbH (UniPark) zur Verfügung gestellt. Diese Firma hat sich auf die sichere Erhebung von Daten im Bereich der Forschung spezialisiert und ist mit ISO 27001 durch das Bundesamt für Sicherheit in der Informationstechnik zertifiziert. Die Übermittelung der Antworten der Befragten zur Fragebogenplattform erfolgt mit SSL-Verschlüsselung. Zusätzlich haben zu keiner Zeit Unbefugte Zugang zu den Datenverarbeitungssystemen und können dementsprechend nicht durch diese eingesehen, gelesen, kopiert, verändert oder entfernt werden.

b) Erhebung über die „Aumio App“

Über die „Aumio“ App werden keine sensiblen, personenbezogenen Daten erfasst. Es werden lediglich Daten erhoben, die zum einen technischen Hintergrund haben (wie z.B. Gerätekennung und -informationen, Sprache, Betriebsversion etc.) und zum anderen das Nutzer*innen-Verhalten darstellen, also beispielweise wie viele Achtsamkeits-Sessions absolviert worden sind oder wie lange die App genutzt wurde. Darüber hinaus werden alle diese Daten in der „Aumio“ App nur unter einem Pseudonym, welches die Teilnehmenden in einer separaten E-Mail erhalten haben, erfasst.

**Speicherung von Daten**

a) Fragebögen über Unipark

Der Zugriff auf die beantworteten Fragebögen, welche Daten über den Schlaf Ihrer Kinder, damit verbundenen psychischen Problembereichen, ihrer Belastung und sozioökonomischer Daten beinhaltet, ist nur passwortgeschützt über die Seite der Questback GmbH möglich. Nur Befugte, also das Studienteam, haben einen passwort-gesicherten Zugang. Nach Beendigung der Datenerhebung werden die Daten von der Seite gelöscht und ausschließlich lokal auf den Rechnern des Studienteams, sowie auf einer externen Festplatte (als Backup), gespeichert. Die Antworten aus den Fragebögen enthalten zudem keine Klarnamen und sind nur über eine Kennziffer zuordenbar. Die Zuweisungsliste, in der die Klarnamen bzw. Mailadressen der Teilnehmenden und die dazugehörigen Kennziffern stehen, werden nur für das Studienteam zugänglich, verschlüsselt und passwortgesichert, lokal auf einem separaten USB-Stick abgespeichert. Die Zuweisungsliste wird also separat von den Daten aufbewahrt, sodass keine Unbefugten, die Daten mit den Klarnamen bzw. der Mailadresse in Verbindung bringen können.

b) Erhebung über die „Aumio“ App

Die Daten, die über die „Aumio“ App erhoben werden, sind nur für die Administrator*innen Aumios verfügbar und werden von diesen an das Studienteam übergeben. Die Aumio UG (haftungsbeschränkt) agiert ebenfalls unter allen geltenden Datensicherheits- und Datenschutzgesetzen, inklusive der geltenden EU-Datenschutzvorschriften. Der Transfer dieser Daten erfolgt ebenfalls über einen passwort-gesicherten USB-Stick, auf dem die Daten wieder verschlüsselt vorliegen werden.

**Auswertung**

Die Zuordnung der Daten aus Unipark und der Daten aus der „Aumio“ App erfolgt über

eine Zuordnungsliste. Diese ist passwort-gesichert, verschlüsselt und nur dem Studienteam zugänglich. Die gewonnen Daten werden zusammengeführt und dann anonymisiert ausgewertet. Es sind keine Rückschlüsse auf einzelne Personen möglich. Die Daten werden nur im Gesamten, nicht in einzelnen kleineren Gruppen ausgewertet, sodass auch so keine Rückschlüsse möglich sind. Zusätzlich sind die Daten in dieser Form auch nur dem Studienteam zugänglich. Alle Daten werden nur lokal bzw. auf einer Festplatte (als Back-up) passwortgesichert gespeichert. Das benötigte Passwort ist nur dem Studienteam zugänglich. Das Studienteam verpflichtet sich zur Schweigepflicht über personenbezogene Daten und zur Einhaltung des Bundesdatenschutzgesetzes. Es werden keine Angaben zu Ihrer Person an Dritte weitergegeben oder veröffentlicht.

**Folgende Rechte haben Sie nach der Datenschutzgrundverordnung (DS-GVO):**

• Auskunft über Verarbeitung personenbezogener Daten (Art. 15)

• Widerruf einer erteilten Einwilligung (Art. 7)

• Berichtigung (Art. 16)

• Löschung (Art. 17)

• Einschränkung der Verarbeitung (Art. 18)

• Recht auf Beschwerde bei einer Aufsichtsbehörde (Art. 77)

Das Einfordern eines der oben genannten Rechte wird keine Negativfolgen für Sie haben.

**Löschung von Daten**

Nach der DS-GVO haben Sie jederzeit das Recht, die Löschung Ihrer Daten einzufordern. Ansonsten beträgt die Aufbewahrungszeit der anonymisierten Daten 10 Jahre. Personenbezogene Daten werden unverzüglich gelöscht, sobald Sie nicht mehr zur Durchführung der Studie erforderlich sind (d.h. nach Abschluss der Studie). Das heißt für unserer Studie konkret, dass wir Ihre personenbezogenen Daten nur solange speichern, wie Sie an der Studie teilnehmen und darüber hinaus, bis eine Auswertung der Daten im Rahmen unserer Forschungsfragen stattfinden konnte. Wir setzen uns demnach selber eine Speicherfrist von einem Jahr. Anschließend werden Ihre Daten von uns gelöscht.

Sie erreichen den verantwortlichen Datenschutzbeauftragten der Freien Universität Berlin, Dr. Karsten Kinast, unter: datenschutz@fu-berlin.de

## E-Mails an die Teilnehmenden während der Studie

*Rückmeldung/Einladungen über Unipark*

**Rückmeldung nach Beendigung der 1. Umfrage (T0)**

**Betreff: Aumio-Studie: Bestätigung zum Ausfüllen der Umfrage**

Sehr geehrte*r Teilnehmer*in,

vielen Dank für das Ausfüllen der Fragebögen. Wir werden Ihre Angaben nun auswerten und Ihnen in den nächsten Tagen eine Rückmeldung bezüglich des weiteren Verlaufes innerhalb unserer Studie geben.

Mit freundlichen Grüßen

Nadja Kristin Ruckser

**Rückmeldung bei Nicht-Erreichen der Einschlusskriterien der Studie**

**Betreff: Aumio-Studie: Rückmeldung zum Studienein-/ausschluss**

Sehr geehrte*r Teilnehmer*in,

es tut uns leid, Ihnen mitteilen zu müssen, dass die Ergebnisse der Fragebögen leider nicht den Einschlusskriterien unserer Studie entsprechen. Dies bedeutet nicht, dass Ihre Belastung oder Ihr Bedarf nicht ernstzunehmend ist, weshalb wir Ihnen folgende Links empfehlen würden, falls Sie sich näher mit der Thematik auseinandersetzen möchten:

*Informationen zu kindlichen Schlafproblemen*

https://www.dgkj.de/eltern/dgkj-elterninformationen/elterninfo-kind-schlaeft-nicht

https://psychologische-coronahilfe.de/beitrag/schlafstoerungen-bei-kindern-und-jugendlichen/

https://www.kindergesundheit-info.de/themen/schlafen/schlafprobleme/unterstuetzung-und-hilfe/

https://www.kindergesundheit-info.de/themen/schlafen/schlafprobleme/haeufige-probleme/

*Hilfe finden:*

https://www.psychenet.de/de/hilfe-finden/schnelle-hilfe/krisenanlaufstellen-kj.html

Bei Rückfragen stehen wir Ihnen gerne unter der E-Mail-Adresse n.ruckser@fu-berlin.de zur Verfügung.

Mit freundlichen Grüßen

Nadja Kristin Ruckser

**Rückmeldung bei Erreichen der Einschlusskriterien der Studie**

**Betreff: Aumio-Studie: Rückmeldung zum Studienein-/ausschluss - Einladung zur Umfrage**

Sehr geehrte*r Teilnehmer*in,

wir freuen uns, Ihnen mitteilen zu können, dass die Ergebnisse der Fragebögen den Einschlusskriterien unserer Studie entsprechen und wir Ihr Kind und Sie in unsere Studie einschließen. Der nächste Schritt ist nun, einen weiteren Online-Fragebogen auszufüllen. Diesen finden Sie unter folgendem Link:

***www.unipark.xyw/123***

In dieser Umfrage sollen Sie die Fragen wieder alleine, ohne Ihr Kind, beantworten. Dies wird ca. xx Minuten beanspruchen. Nach Abschluss des Fragebogens erhalten Sie eine weitere E-Mail von uns, in der wir Ihnen mitteilen, ob Sie der Interventionsgruppe angehören und somit gleich die „Aumio“ App erhalten oder ob Sie der Wartegruppe angehören und dementsprechend die App erst nach sechs Wochen erhalten.

Bei Rückfragen stehen wir Ihnen gerne unter der angegeben E-Mail-Adresse zur Verfügung.

Mit freundlichen Grüßen

Nadja Kristin Ruckser

n.ruckser@fu-berlin.de

**Rückmeldung nach Beendigung der 2. Umfrage (T0, Teil 2)**

**Betreff: Aumio-Studie: Code zur kostenfreien Nutzung der Aumio App**

Sehr geehrte*r Teilnehmer*in,

vielen Dank für die Bearbeitung der zweiten Umfrage! Sie und Ihr Kind sind nun Teil unserer Studie. Für die Anmeldung in der App benötigen Sie Ihre Teilnehmer-ID, die Sie in Unipark erstellt haben. Zur Erinnerung ist hier das Schema, nach dem Sie diese erstellt haben:

Xx von xx

Xx von xx

Xx xx

Für den kostenfreien Zugang zur Aumio App geben Sie bitte folgenden Code in der App ein: Xx xx

In der Information für Teilnehmende finden Sie nochmal die Hinweise, wie die App zu nutzen ist. Kurz zusammengefasst: Benutzen Sie „Aumio“ so wie es am besten in den Alltag Ihres Kindes und Ihnen passt. Idealerweise machen Sie mehrmals pro Woche eine Übung, wenn das jedoch nicht klappt, ist es auch nicht so schlimm. Sie können gerne bei den Übungen dabei sein und diese mitmachen, wenn Ihr Kind das möchte.

In sechs Wochen erhalten Sie dann erneut einen Link von uns, der Sie zu einer weiteren Umfrage führt. Es werden die gleichen Fragebögen sein, die Sie schon in der ersten und zweiten Umfrage kennengelernt haben. Wie auch schon bei unserer ersten Befragung werden wir ausschließlich Fragen an Sie stellen.

Falls Sie Fragen haben sollten, können Sie sich gerne bei uns melden.

Mit freundlichen Grüßen

Nadja Kristin Ruckser

**Einladung zu T1**

**Betreff: Aumio-Studie: Einladung zur Umfrage**

Sehr geehrte*r Teilnehmer*in,

wir möchten Sie erneut dazu einladen, unsere Umfrage zu beantworten. Es werden die gleichen Fragebögen sein, die Sie schon kennengelernt haben. Wie auch schon bei unserer ersten Befragung werden wir ausschließlich Fragen an Sie stellen, dieser Teil wird ca. xx Minuten beanspruchen.

Folgend finden Sie den Link zur Umfrage:

***www.unipark.xyw/123***

Bei Fragen können Sie sich gerne bei uns melden!

Mit freundlichen Grüßen

Nadja Kristin Ruckser

**Rückmeldung nach Beendigung von T1**

**Betreff: Bestätigung zum Ausfüllen der Umfrage**

Sehr geehrte*r Teilnehmer*in,

vielen Dank für das erneute Ausfüllen der Umfrage. Sie erhalten erst in sechs Wochen wieder eine Einladung zu einer weiteren Umfrage von uns. Diese umfasst die gleichen Fragebögen, die Sie bereits kennengelernt haben. Wie auch schon bei unserer ersten Befragung werden wir ausschließlich Fragen an Sie stellen. Bis dahin können Sie gerne „Aumio“ weiter nutzen, so wie es Ihnen beliebt.

Bei Fragen können Sie sich gerne bei uns melden!

Mit freundlichen Grüßen

Nadja Kristin Ruckser

**Einladung zu T2**

**Betreff: Aumio-Studie: Einladung zur letzten Umfrage**

Sehr geehrte*r Teilnehmer*in,

es ist wieder soweit: wir möchten Sie erneut dazu einladen, unsere Umfrage zu beantworten. Das Prozedere ist wie gehabt: wir werden ausschließlich Fragen an Sie stellen. Dieser Teil wird ca. xx Minuten beanspruchen. Folgend finden Sie den Link zur Umfrage:

***www.unipark.xyw/123***

Bei Fragen können Sie sich gerne bei uns melden!

Mit freundlichen Grüßen

Nadja Kristin Ruckser

**Rückmeldung nach Beendigung von T2**

**Betreff: Ende der Studie**

Sehr geehrte*r Teilnehmer*in,

vielen Dank für die Bearbeitung der dritten Umfrage! Wir freuen uns, dass Sie so lange mitgemacht haben und am Ball geblieben sind. Auch wenn die Studie für Sie jetzt offiziell beendet ist, können Sie gerne „Aumio“ weiterhin benutzen. Die App bleibt für Sie verfügbar.

Wir wünschen Ihnen weiterhin alles Gute!

Mit freundlichen Grüßen

Nadja Kristin Ruckser

*Reminder T1/T2 (zum Erinnern über Gmail)*

Betreff: Erinnerung Ausfüllen 2./3. Umfrage

Sehr geehrte*r Teilnehmer*in,

vorneweg möchten wir uns bei Ihnen bedanken, dass Sie Teil unserer Studie sind!

Vor Kurzem haben wir Ihnen einen Link zur zweiten/dritten Umfrage zukommen lassen. **Falls Sie diese bereits ausgefüllt haben, können Sie diese E-Mail ignorieren**. Falls Sie die Umfrage noch nicht ausgefüllt haben, möchten wir Sie hiermit erinnern, das bitte noch zu machen.

Uns ist bewusst, dass Sie sicher viel zu tun haben, dennoch ist es aus studientechnischer Sicht sehr wichtig, wenn Sie sich ca. XX Minuten Zeit nehmen, den zweiten/dritten Teil der Studie auszufüllen. Ohne Ihre Angaben zum jetzigen Zeitpunkt, könnten alle anderen Daten nämlich nicht ausgewertet werden. Nur mit den neuen Angaben können wir ermitteln, welche Wirkung die Aumio-App hat.

Falls Sie Fragen haben, können Sie sich jederzeit bei mir unter n.ruckser@fu-berlin.de melden.

Vielen Dank im Voraus!

Mit freundlichen Grüßen

Nadja Kristin Ruckser
